# Visualization of Speech Perception Analysis *via* Phoneme Alignment: A Pilot Study

**DOI:** 10.3389/fneur.2021.724800

**Published:** 2022-01-11

**Authors:** J. Tilak Ratnanather, Lydia C. Wang, Seung-Ho Bae, Erin R. O'Neill, Elad Sagi, Daniel J. Tward

**Affiliations:** ^1^Center for Imaging Science and Institute for Computational Medicine, Department of Biomedical Engineering, Johns Hopkins University, Baltimore, MD, United States; ^2^Center for Applied and Translational Sensory Sciences, University of Minnesota, Minneapolis, MN, United States; ^3^Department of Otolaryngology, New York University School of Medicine, New York, NY, United States; ^4^Departments of Computational Medicine and Neurology, University of California, Los Angeles, Los Angeles, CA, United States

**Keywords:** phoneme alignment, speech tests, phoneme accuracy, relative information transfer, F1-score

## Abstract

**Objective:** Speech tests assess the ability of people with hearing loss to comprehend speech with a hearing aid or cochlear implant. The tests are usually at the word or sentence level. However, few tests analyze errors at the phoneme level. So, there is a need for an automated program to visualize in real time the accuracy of phonemes in these tests.

**Method:** The program reads in stimulus-response pairs and obtains their phonemic representations from an open-source digital pronouncing dictionary. The stimulus phonemes are aligned with the response phonemes via a modification of the Levenshtein Minimum Edit Distance algorithm. Alignment is achieved via dynamic programming with modified costs based on phonological features for insertion, deletions and substitutions. The accuracy for each phoneme is based on the F1-score. Accuracy is visualized with respect to place and manner (consonants) or height (vowels). Confusion matrices for the phonemes are used in an information transfer analysis of ten phonological features. A histogram of the information transfer for the features over a frequency-like range is presented as a phonemegram.

**Results:** The program was applied to two datasets. One consisted of test data at the sentence and word levels. Stimulus-response sentence pairs from six volunteers with different degrees of hearing loss and modes of amplification were analyzed. Four volunteers listened to sentences from a mobile auditory training app while two listened to sentences from a clinical speech test. Stimulus-response word pairs from three lists were also analyzed. The other dataset consisted of published stimulus-response pairs from experiments of 31 participants with cochlear implants listening to 400 Basic English Lexicon sentences via different talkers at four different SNR levels. In all cases, visualization was obtained in real time. Analysis of 12,400 actual and random pairs showed that the program was robust to the nature of the pairs.

**Conclusion:** It is possible to automate the alignment of phonemes extracted from stimulus-response pairs from speech tests in real time. The alignment then makes it possible to visualize the accuracy of responses via phonological features in two ways. Such visualization of phoneme alignment and accuracy could aid clinicians and scientists.

## Introduction

**Figure 1:**
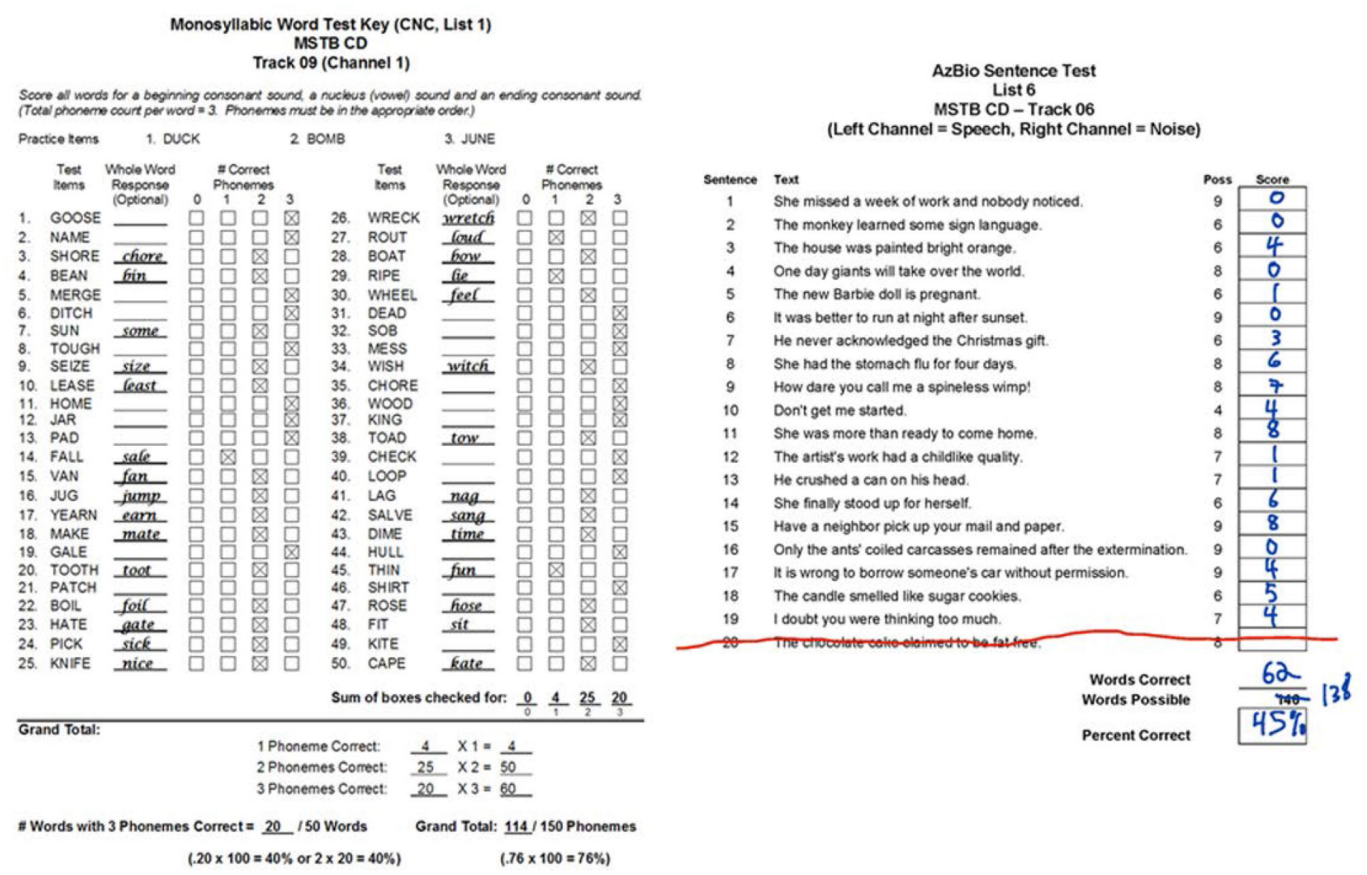
Two examples of typical speech perception tests performed in the clinic. Both lists were obtained from the new Minimum Speech Test Battery (MSTB) for Adult Cochlear Implant Users [Auditory Potential LLC, (9)]. The one on the left (from page 12 of MSTB manual, https://www.auditorypotential.com/MSTB_Nav.html) shows the actual results from 50 monosyllabic consonant-nucleus-consonant (CNC) words. The clinician records the incorrect response and the number of correct phonemes for each stimulus. A tally of the number of correct words and phonemes is presented. The one on the right (from page 6 of the MSTB score sheets, https://www.auditorypotential.com/MSTB_Nav.htm) shows the results from a volunteer (see Methods - Datasets) listening to 19 sentences from the AzBio list #6. The clinician records the number and the total percentage of correct sentences. See Figure 6 for the corresponding visual representation of these scores.

Audiologists and speech pathologists use speech perception tests to analyze speech comprehension in people who are learning to hear with hearing aids and cochlear implants. Specifically, the tests provide an objective measure of how the listener processes spoken words and sentences from the acoustic signal. The words and sentences are composed of sequences of phonemes that are characterized as either consonants or vowels. Further, phonemes are differentiated by how they are produced in the vocal tract, i.e. phonological features (1). For consonants, these features are place, manner, voicing and associated subtypes, and for vowels, these are height, place and associated subtypes. Typically, speech tests are based on lists of words or sentences and are presented in a sound booth in the clinic, sometimes with noise, e.g. PBK-50 (2), AB (3), NU-6 (4), BKB (5), CUNY (6), HINT (7) and AzBio (8). Usually, the clinician records the numbers of correct words and/or sentences, and sometimes the number of correct phonemes, as illustrated by two examples in Figure 1. One example is a typical list of 50 words, each with an initial consonant followed by a nucleus (vowel) and then a final consonant. Here, the correct response and number of correct phonemes are recorded. The result is a tally of the number of correct words and phonemes together with incorrect words transcribed. The other example is a typical list of 20 phonetically balanced sentences. Here, the number of correct words is recorded and then summed. It is clear in both cases that the person does not always hear the whole stimulus. There is potentially more useful data to be extracted from these tests, namely the analysis of phonemes with respect to their phonological features. To do so in the clinic would be time consuming. The challenge then is to present information about phonemic comprehension in a manner that can be understood in real time.

At the same time, many people learning to hear with a new hearing aid or a cochlear implant use auditory training apps such as the Speech Banana app which is freely available (10). Progress tracking provides the user a record of correct sentences, correct words, and number of repetitions in the quizzes. Additional information such as accuracy for the phonemes could help the user work remotely or in person with the clinician to identify areas of weaknesses. To that end, visualization of phonemic accuracy could be useful as motivation and diagnostic tool for patient and clinician respectively especially in the telemedicine era.

Hence, there is a need for an automated program to compute and visualize the accuracy of phonemes from responses to speech stimuli in real time. Specifically, given a stimulus-response pair of words or sentences, the problem is to develop and implement the automated program in four steps. First, use an online pronunciation dictionary to express the stimulus and response as two ASCII sequences of phonemes. Second, use an alignment technique to align the sequences. Third, calculate and visualize phoneme accuracy with respect to phonological features and associated subtypes. Fourth, make the program available to the computational audiology community.

The first two steps can be accomplished by leveraging two tools commonly used in speech recognition research. For the first, there are several online pronunciation dictionaries: Pronlex, CMUDict, CELEX and UNISYN to name but a few (11). Of these, CMUDict is publicly available and has been widely used in open source automatic speech recognition software such as Kaldi (12). For the second, several sequence alignment algorithms are available from scLite, which is part of an open source library (13, 14). The third step makes use of two commonly used metrics: a F1-score (Sørenson-Dice coefficient) for the phonemes and relative information transfer for the phonological features. The fourth step deploys the program in MATLAB so that it can be converted for open-source usage.

Using a pronunciation dictionary followed by automated sequence alignment for analyzing speech comprehension by people with hearing loss is not new. Previous uses include analyses of lipreading by people with normal hearing and hearing loss (15–18), estimating intelligibility from atypical speech (19–21) and more recently, listening to speech in noise by people with normal hearing (22). Using relative information transfer to analyze speech comprehension based on phonological features of transcribed phonemes is also not new. In addition to Bernstein (15), previous uses include analyses of listening by people with hearing loss (23–31). There was also a study of bimodal hearing with hearing aid and cochlear implant that manually transcribed phonemes with the aid of a digital dictionary (32). The approach here differs from earlier work in that the program is made publicly available by adopting and modifying two open-source algorithms and two commonly used metrics, with the goal of providing a visual representation of results similar to those shown in Figure 1.

This paper describes a pilot study of the design and implementation of the automated program. It reports the program's validation and the results of using it in several cases. Finally, it discusses the advantages and disadvantages of the program and provides suggestions for clinical usage.

## Methods

This section describes: (i) the design of the program; (ii) how stimulus-response pairs of words or sentences are formatted as two sequences of phonemes; (iii) how two sequences are aligned; (iv) how the F1-score is used to compute the accuracy of the stimulus phonemes; (v) how relative information transfer is used to assess the accuracy based on phonological features; (vi) how the preceding two metrics can be visualized for a set of stimulus-response pairs; (vii) the different datasets used for testing; and (viii) program validation.

### Design

**Figure 2:**
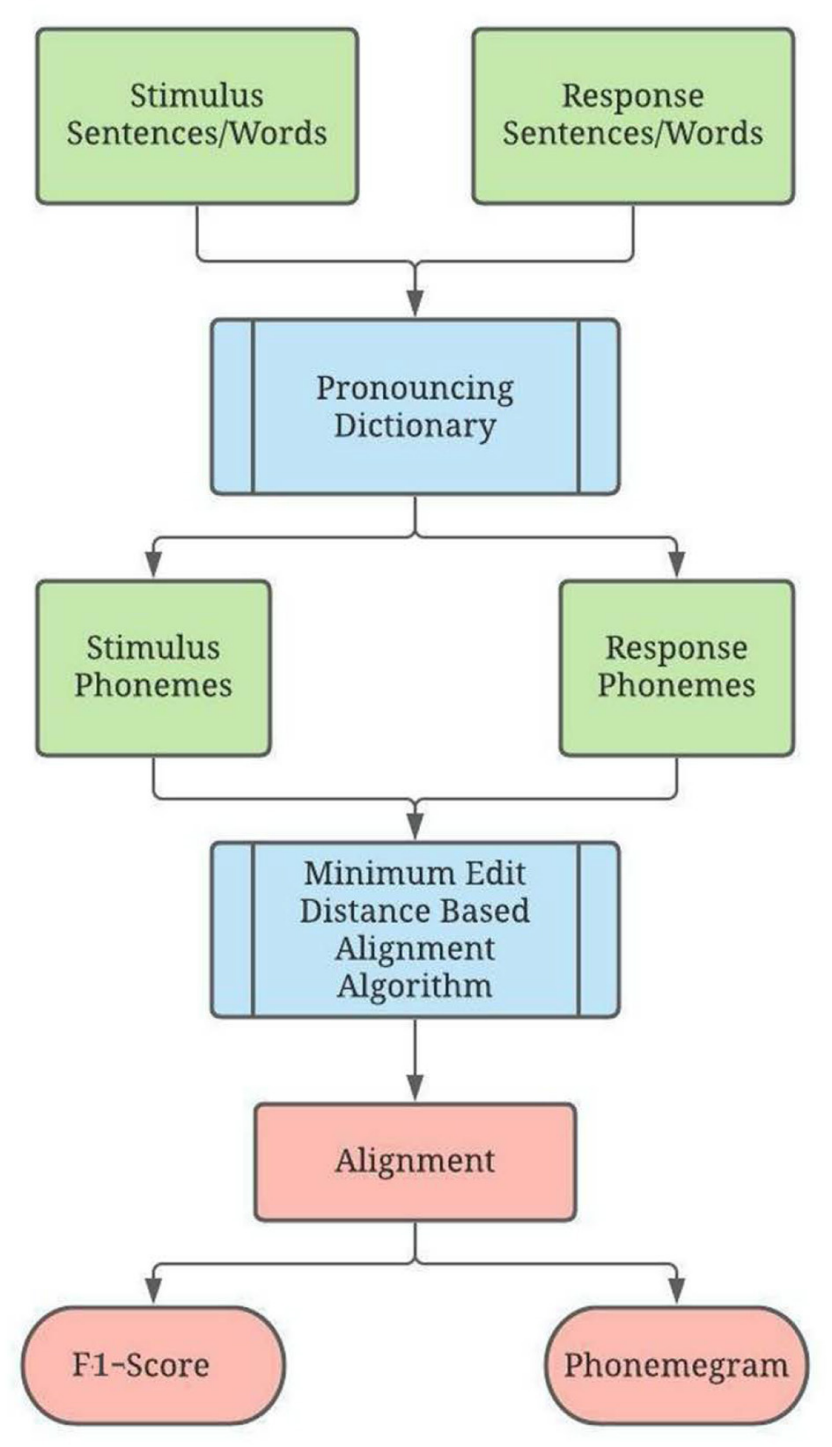
Program Overview. Here, green represents input, blue represents the core algorithm, and red represents output. The program takes a set of stimuli and the set of corresponding responses as parameters. The stimuli and responses are translated from words into phonemes using a digital pronouncing dictionary. The phonemes for each stimulus-response pair are passed into the alignment algorithm, which displays an alignment and phoneme accuracy. Once all stimulus-response pairs have been evaluated, graphics of phonemegram and phoneme accuracy for vowels, voiced and unvoiced consonants are generated.

Figure 2 illustrates the overall design for analyzing the response of a person with hearing loss listening to sentences or words in speech tests in real time. The program first takes as input stimulus-response pairs in the form of sentences or words. Both are converted to phonemes using a digital pronunciation dictionary for each word, and the phonemes are entered into the alignment algorithm. Then the accuracy for the stimulus phonemes is computed in two ways via a F1-score for each phoneme and relative information transfer for ten different phonological features.

### Input

The program uses the Carnegie Mellon University Pronouncing Dictionary (CMUDict) which is an open-source machine-readable pronunciation dictionary for North American English that contains over 134,000 words and their pronunciations (33). CMUDict has been widely used (11) for speech recognition and synthesis, as its entries map words to their pronunciations as ASCII symbols in the ARPAbet format (34). The ARPAbet format contains 39 phonemes with vowels each carrying a lexical stress marker. Transcriptions are expressed as strings of phonemes. The raw text file for the most stable version of CMUDict (0.7b) was downloaded from http://www.speech.cs.cmu.edu/cgi-bin/cmudict, and saved as a MATLAB map data structure. Also, lexical stress markers were removed as they did not affect the subsequent analysis. Misspelled or incorrectly pronounced words, however, need to be modified by the user. For example, in a YouTube demonstration of a subset of PBK-50, the word “pinch” was misheard as “kints,” which is a nonsense word. Since CMUDict is not able to translate “kints” into phonemes, the user is directed to the online dictionary and enters real words such as “mints” and “key” yielding “M IH N T S” and “K IY” respectively so that “K IH N T S,” is manually entered as the phonemic representation of “kints”. Since the program splits its input sentences into words, it only requires manual input for nonsense words, not the entire sentence containing them.

### Alignment

Given a paired strings of phonemes for the stimulus and response, the next step is to align the phonemes. Algorithms for aligning strings arise in other areas including bioinformatics (11, 35). The goal is to minimize the distance between two strings. The minimum edit distance (MED), based on the classic Levenshtein distance algorithm (36), computes the number of editing operations (insertion, deletion, and substitution) needed to transform one string to the other. Each operation is associated with a numerical cost or weight. Here the costs are modified for the particular case of aligning strings of phonemes. The MED is computed by applying dynamic programming (35) to generate an edit distance matrix which is a table of transitions from one string to the other. A global solution is built by solving and remembering the solutions to simpler subproblems, resulting in an alignment with the minimum associated cost.

The first version of alignment was implemented in MATLAB and used the Levenshtein algorithm from scLite (14) that used simple costs−1 for insertion or deletion, 2 for substitution, and 0 for a match. These favored quick matches, sometimes aligning a response phoneme far from any others—for example, if “bite” was the response to the stimulus of “birds bite,” the initial “B” consonant in “bite” was aligned with that in “birds”. As this caused issues with longer sentences, the costs were modified to discourage switching from a deletion (a space within the aligned response) to an insertion or substitution (of a response phoneme), and vice versa. As a first step toward avoiding multiple alignments, modification was accomplished by adding a 0.5 cost for deletion if the previous operation was insertion or substitution, and a 0.5 cost for insertion/substitution if the previous operation was deletion. The two exceptions are within the substitution cost. To favor matches, the cost for a match after a deletion or an insertion is an extra 0.2 or 0.1, respectively, instead of 0.5. These costs are summarized in the left half of the first row and the second and third columns of **Table 2**.

Initially, the algorithm was coded to generate one alignment once the edit distance matrix was filled. However, this did not guarantee the best alignment. Multiple alignments led to the same MED if, for example, fewer phonemes than expected were entered, and the algorithm aligned incorrect phonemes in different places. Previously, the algorithm would assign each cell in the edit distance matrix a single operation, even if two or more operations led to the same MED. Consequently, the algorithm would generate a single alignment, arbitrarily based on the order of costs evaluated. By logging all of the operations that led to the same MED in a cell of the edit distance matrix, this single alignment was found to be a result of these simple costs. Many alignments—even over 1000—led to the same MED. The costs were then modified to favor substitutions for phoneme alignments that have similar phonological features (1). Table 1 maps the following 10 phonological features to the 39 phonemes: nasality, vowel height, manner, voicing, contour, vowel place, vowel length, affrication, sibilance and consonant place. These features and their subtypes (described in the caption for Table 1) are used to deem consonant-consonant and vowel-vowel alignments sharing all or most of their attributes as similar, and given a substitution cost deduction to favor substitution of “similar” phonemes. For example, a voiced “F” results in a “V”, so the program will prefer the substitution of these two phonemes over any other incorrect substitutions. Even after implementing the similarity cost deductions, the algorithm often generated several alignments, some of which were preferable to others. To further favor alignments that represent probable errors, a slight consonant manner cost deduction was implemented, in order to prefer substitution between two manner subtypes such as stops, fricatives, or glides. For example, if the algorithm must choose between aligning the stop phoneme “K” with the fricative “S” or the stop “P”, the algorithm will choose to align the stops together. Details of possible consonant-consonant, vowel-vowel and consonant manner pairs are given in the Supplementary Data. Last but not least, vowel-consonant substitution is heavily penalized to prevent alignment of consonants with vowels. With these modified costs, the alignment should then accurately reflect the response. These costs are summarized in Table 2.

Table 1Phonological features of consonants and vowels based on Ladefoged and Johnstone (1).

**Table:** 

**Phonological Features (Vowels)**				
**Phoneme**	**Vowel height**	**Contour**	**Vowel place**	**Vowel length**
AA	0	1	2	0
AE	0	1	1	0
AH	1	1	1	0
AO	1	1	2	0
AW	0	2	1	1
AY	0	0	1	1
EH	1	1	0	0
ER	1	1	0	0
EY	1	1	1	0
IH	1	0	0	1
IY	2	1	0	0
OW	1	0	2	1
OY	1	2	2	1
UH	2	1	2	0
UW	2	1	2	1

**Table:** 

**Phonological Features (Consonants)**						
**Phoneme**	**Nasality**	**Manner**	**Voicing**	**Affrication**	**Sibilance**	**Place**
B	0	0	1	0	0	0
CH	0	4	0	1	1	1
D	0	0	1	0	0	1
DH	0	2	0	1	0	0
F	0	2	0	1	0	0
G	0	0	1	0	0	2
HH	0	2	0	1	0	2
JH	0	4	1	1	1	1
K	0	0	0	0	0	2
L	0	3	1	0	0	1
M	1	1	1	0	0	0
N	1	1	1	0	0	1
NG	1	1	1	0	0	2
P	0	0	0	0	0	0
R	0	3	1	0	0	1
S	0	2	0	1	1	1
SH	0	2	0	1	1	1
T	0	0	0	0	0	1
TH	0	2	1	1	0	0
V	0	2	1	1	0	0
W	0	3	1	0	0	0
Y	0	3	1	0	0	1
Z	0	2	1	1	1	1
ZH	0	2	1	1	1	1

*Values of subtypes for vowel height are: 0 = low, 1 = mid, 2 = high; vowel place are 0 = front, 1 = central, 2 = back; contour are 0 = rising, 1 = flat, 2 = falling; vowel length are 0 = short, 1 = long; consonant manner are: 0 = stop, 1 = nasal; 2 = fricative; 3 = glide; 4 = affricate and consonant place are: 0 = front, 1 = center, 2 = back*.

**Table 2:** Costs for each operation (left–insertion and deletion; right–substitution), depending on the previous operation.

**Current operation**	**Insertion**		**Deletion**		**Current operation**	**Substitution**		
Previous operation	Ins	Sub/Del	Del	Ins/Sub	Previous operation	Sub	Ins	Del
Cost	1	1.5	1.5	1	Vowel-cons or vice versa	5	5.1	5.5
					Consonant-consonant	1.75	1.85	2.25
					Same manner consonants	1.3	1.4	1.8
					Similar consonants	1.2	1.3	1.7
					Vowel-vowel	0.9	0.8	1.4
					Similar vowels	0.65	0.75	1.15
					Match	0	0.1	0.2

*The previous operation is considered in order to prefer alignments with the fewest phoneme-to-space and space-to-phoneme transitions. The left half of the first row shows the addition of a 0.5 cost for deletion if the previous operation was insertion or substitution, and a 0.5 cost for insertion/substitution if the previous operation was deletion. The first column on the right shows the costs for the substitutions. The second and third columns show the two exceptions for the substitution cost—to favor matches, the cost for a match after a deletion or an insertion is an extra 0.2 or 0.1. See Supplementary Data for examples of similar consonants, same manner consonants, and similar vowels*.

**Figure 3:**
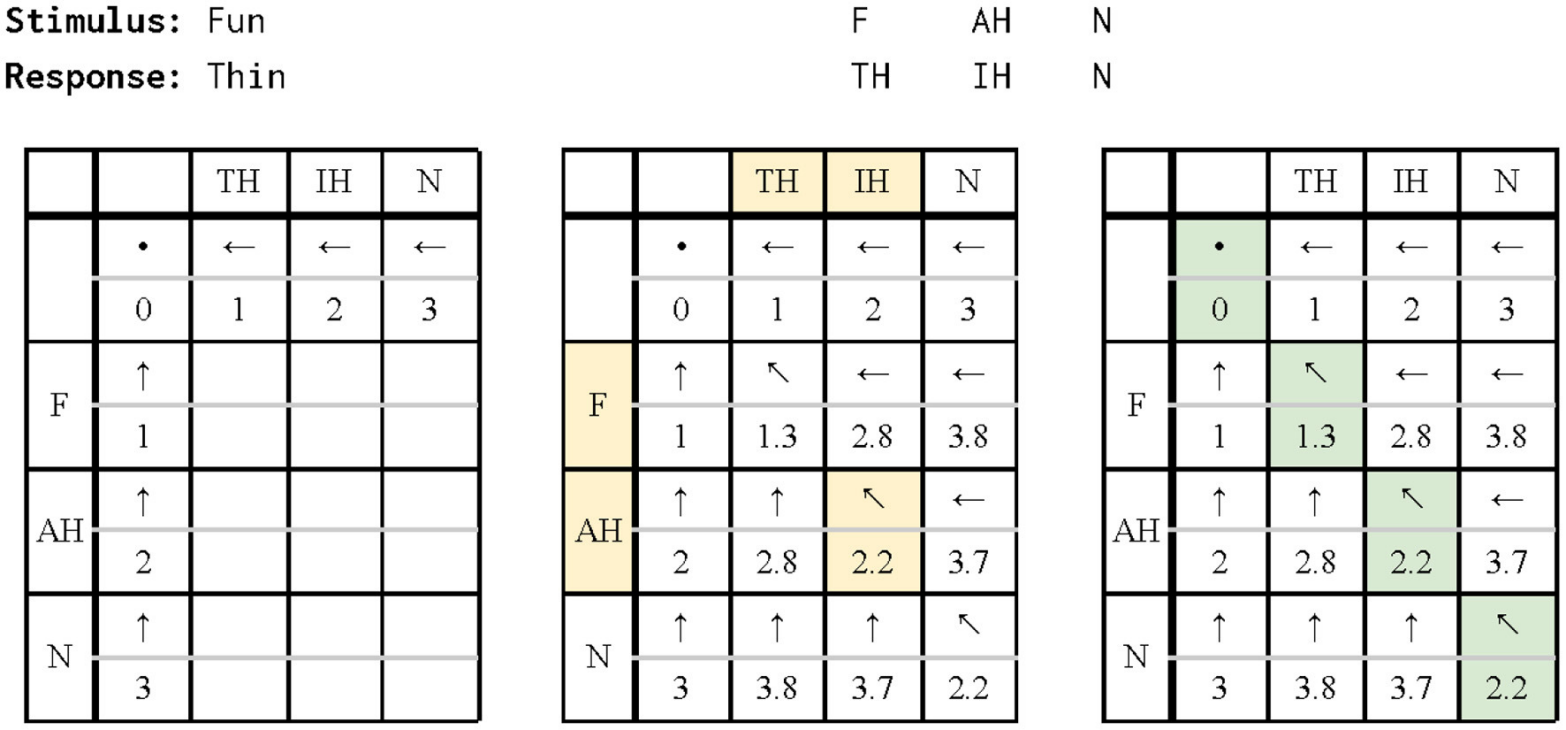
The response phonemes are placed on the top row of the edit distance matrix, while the stimulus phonemes are on the left column. Each square represents the minimum edit distance (MED) for the substrings on each axis, and shows what operation was executed to get to that MED (← is insertion, ↑ is deletion, ↖ is substitution). **Left**: These squares (comparing all substrings of response or stimulus sentence to an empty string) are filled in first, to provide base cases for the rest of the matrix. The MED between an empty string and any string of length *n* is equal to *n*. **Middle**: The highlighted square finds the MED between the response of “TH IH” and the stimulus of “F AH.” It does this by building on the squares of the matrix that have already been filled. Insertion entails aligning the IH with a space (cost 1.5) and adding onto the optimal alignment of “TH” and “F AH” (cost 2.8), for a total cost of 4.3; deletion aligns a space with the AH (1.5) and adds onto the alignment of “TH IH” and “F” (2.8), for a total cost of 4.3; substitution aligns the IH with the AH (0.9) and adds to the alignment of “TH” and “F” (1.3), for a total cost of 2.2. The substitution cost is the lowest, so the matrix records the cost of 2.2 and the substitution operation. **Right**: Once the entire matrix has been filled, the algorithm finds how it generated the MED by tracing back the recorded operations. In this case, the MED of “TH IH N” and “F AE N” is 2.2, and the alignment consists of three substitutions.

**Figure 4:**
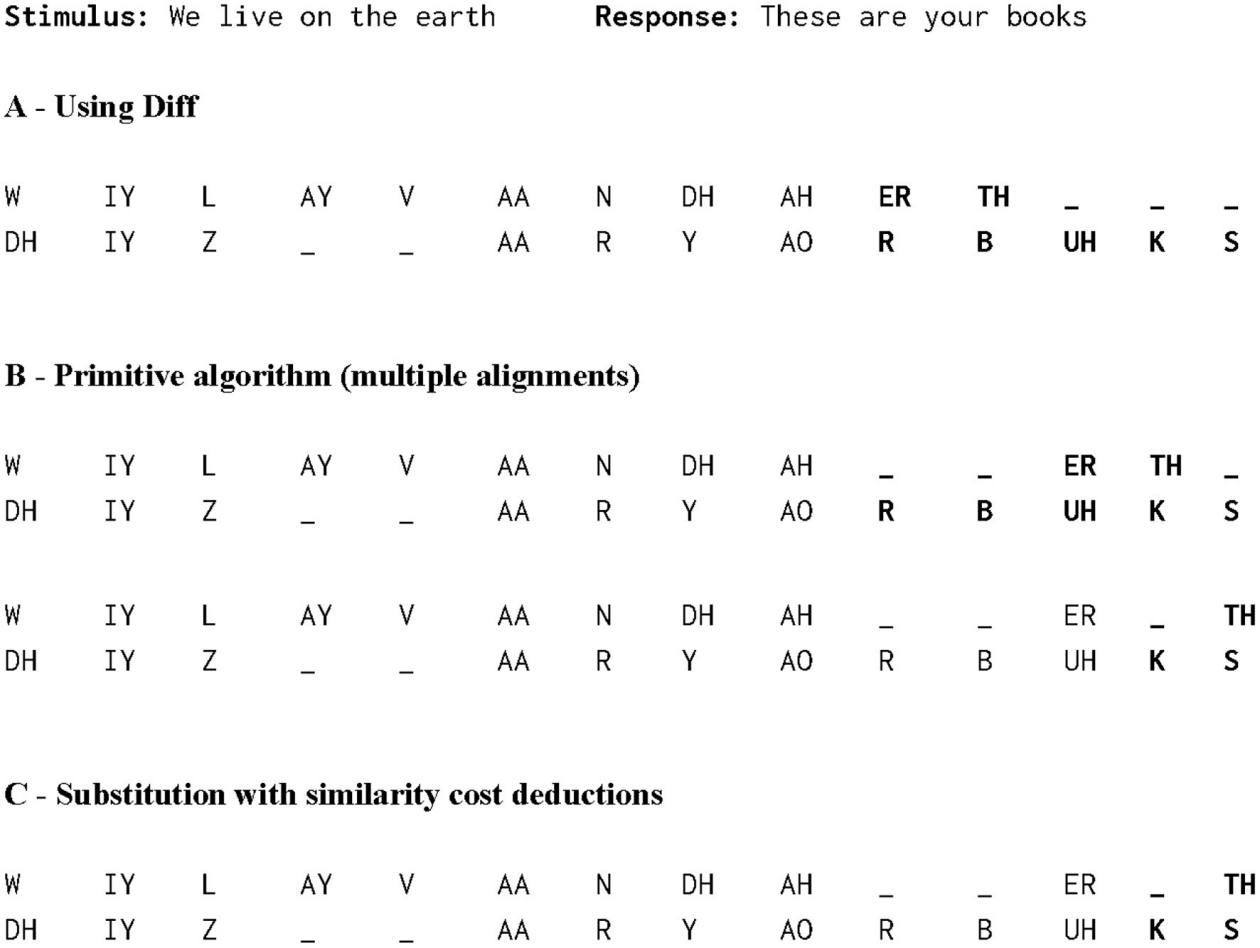
Comparison of three different alignment algorithms for a stimulus-response pair taken from test data V1-HA (see Table 3). **(A)** The alignment generated by the UNIX diff function. The function gives no weight to consonants or vowels, and has no issues with aligning consonants with vowels and vice versa, as shown by the bolded area. **(B)** Multiple alignments generated by primitive algorithm, with no similarity substitution costs. Although the most of the response matches the stimulus, the algorithm generated two alignments with the same MED. **(C)** With the similarity substitution cost implemented, the algorithm generates only one alignment, because S and TH are produced in a similar manner, and therefore have a substitution cost deduction.

Figure 3 shows an example of the operations used in MED with the costs from Table 2 for aligning the response “thin” with the stimulus “fun”. Figure 4 shows the differences between the outputs for aligning the response “We live on the earth” with the stimulus “These are your books” (from participant V1-HA in the test data, see below and Table 3) from three different alignments: the diff function first used in UNIX (37) and available in scLite, the original Levenshtein algorithm with simple costs, and the finalized modified algorithm. diff gave no weight to consonants or vowels. The unmodified algorithm yielded two alignments generated with no similarity substitution costs whereas the modified algorithm with similarity substitution yielded just one alignment, because “S” and “TH” are two consonants with similar manner that are assigned a substitution cost deduction.

**Table 3:** Number of sentence or word stimuli and their responses with the program run time for the examples shown in Figures 6–11 and Supplementary Figures 1–4.

**Participant**	**Figure**	**Stimuli dataset**	**# Stimulus sentences**	**# Correct response sentences**	**# Stimulus words**	**# Correct response words**	**# Stimulus phonemes**	**# Response phonemes**	**Time (secs)**
V1 - CI+HA	7	SB	30	13	165	122	490	483	6.5
V1 - CI	7	SB	30	12	162	101	484	446	4.3
V1 - HA	7	SB	30	0	160	36	488	317	4.0
V2 - CIC HA	S1	SB	30	28	164	159	470	473	3.5
V2 - No HA	8	SB	30	11	153	66	458	211	3.3
V3 – HA	8	SB	30	9	160	108	488	378	3.5
V4 – HA	S1	SB	30	27	164	158	470	473	3.3
V5 – CI	S1	AzBio#1	19	11	146	128	527	515	3.3
V6 – CI	6	AzBio#6	19	3	138	64	492	396	3.2
Anonymous	6	CNC			50	23	150	148	3.0
Anonymous	9	PBK			25	7	69	82	7.0
Several	9	AB			38	0	114	118	2.8
Actual (*N* = 31)	10,11, S2-S4	BEL	12,400	4,310	74,245	43,514	281,480	212,584	364.5
Random (*N* = 31)	11	BEL	12,400	3	74,245	7,424	281,480	212,584	358.8

*CI, cochlear implant; HA, Hearing Aid; CIC, Completely in Canal; SB, Speech Banana; AB, Boothroyd*.

### F1-Score

**Figure 5:**
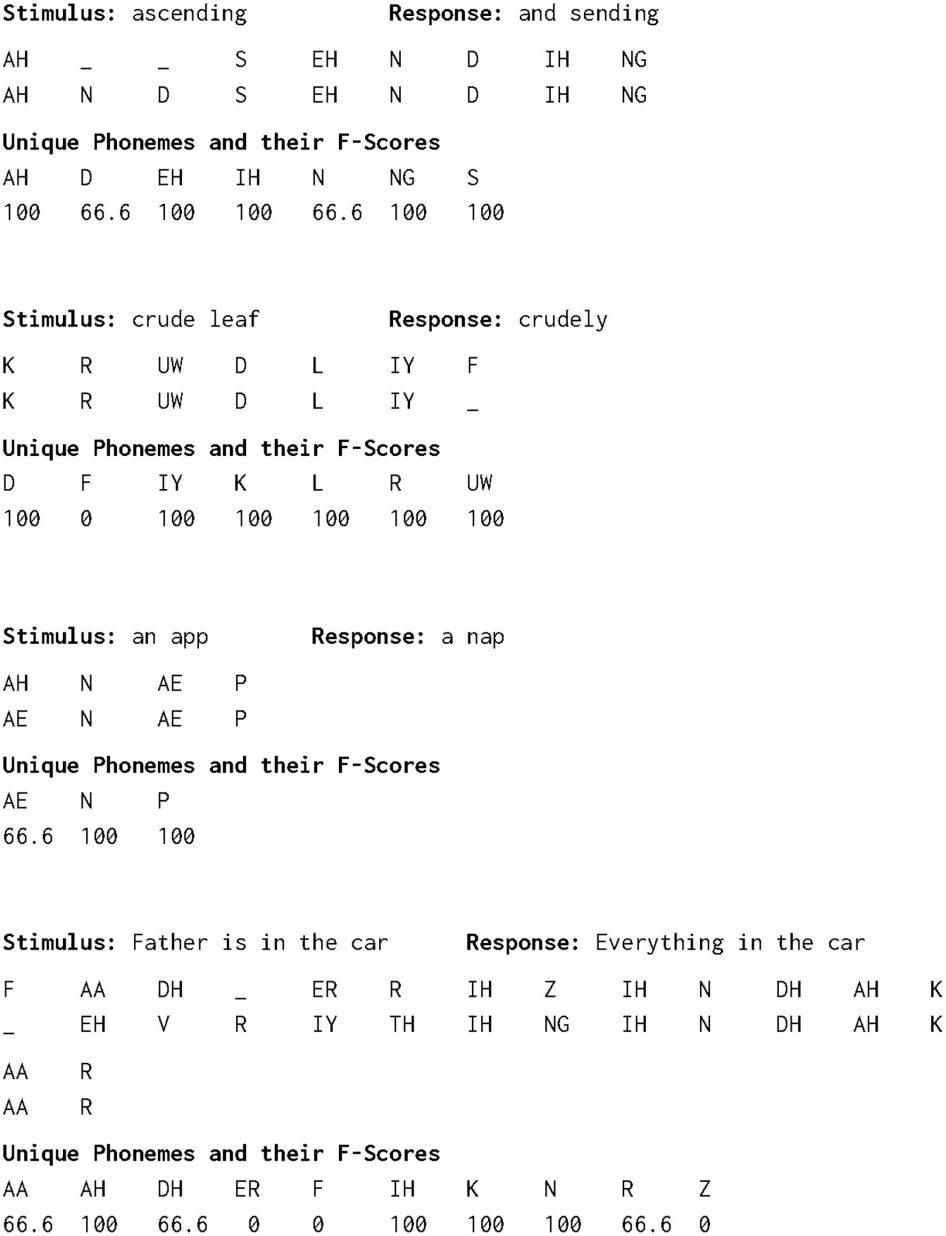
Four examples of alignments and phoneme percent accuracy. The first example shows insertion of the phonemes N and D. The second example shows deletion of the phoneme F. The third example shows substitution of the AE phoneme (æ) for the AH phoneme (ə). The fourth example has all three minimum edit distance operations within its alignment.

For each phoneme in each stimulus, the true positive (*TP*), false positive (*FP*) and false negative (*FN*) values were used to compute the F1-Score, or the Sørensen-Dice coefficient, which is defined as the harmonic mean of precision (*TP*/(*TP* + *FP*)) and sensitivity (*TP*/(*TP* + *FN*)), i.e., 2*TP*/(2*TP* + *FP* + *FN*). Consider the phoneme “K” as an example. A *TP* occurs when a “K” response is matched with a “K” stimulus; a *FN* occurs when not recording a “K” stimulus; a *FP* occurs when recording a non-existent “K”. Figure 5 shows examples of alignments and phoneme F1-scores for four challenging stimulus-response pairs. The first two are examples of the consequences of insertion and deletion [see Table 4 from (38)]. The third example is one of phonemic ambiguity but with different alignments caused by one substitution. The fourth illustrates the use of all three MED operations in the alignment.

### Phonemegram

Following ideas by Danhauer and Singh (29–31), Blamey et al. (25) and others (15, 32, 39–41), an alternative way of visualizing speech comprehension performance is to construct a phonemegram. Specifically, a histogram of relative information transfer for the phonological features from Table 1 over a range from low to high frequency was created as follows. First, confusion matrices for the consonants and vowels were generated. Each matrix consisted of *N* rows of phonemes in the stimulus set and *N* + 1 columns of phonemes in the response set with the extra column reserved for unclassified phonemes due to empty responses (40, 42). The matrices were regenerated as several smaller ones based on the prescribed phonological features. For example, within the vowel height feature, vowels can be further divided into three separate categories: high, mid, and low. In this way, the relative information transfer can be obtained for different features. Following Miller and Nicely (43) and others, the information transfer for each feature was computed via *IT* = log(*n*) + *H*_*x*_ + *H*_*y*_ − *H*_*xy*_ where *H*_*x*_, *H*_*y*_, and *H*_*xy*_ refer to the row (stimulus), column (response), and element entropy respectively, while *n* refers to the total number of entries within the feature matrix. The entropies are characterized by:


$$\begin{array}{l} H=\frac{1}{n}\sum s\;\log\left(s\right) \end{array}$$

where *s* refers to either the individual elements, row sums, or column sums of the feature matrix for computing *H*_*xy*_, *H*_*x*_, and *H*_*y*_ respectively. Then the relative information transfer is given by:


$$\begin{array}{l} H_{stim}=\;-\overset{n}{\underset{i=1}{\sum }}\left(\frac{p_{i}}{p_{total}}\right)\log\left(\frac{p_{i}}{p_{total}}\right) \end{array}$$

where *n* refers to the number of different sub-categories within the feature, *p*_*i*_ refers to the number of phonemes presented that are within the given sub-category, and *p*_*total*_ refers to the total number of phonemes (regardless of subcategory). For example, if out of 16 consonants presented, seven are voiced and nine are unvoiced, then *H*_*stim*_ = −(7/16)log(7/16) − (9/16)log(9/16).

### Output

For each stimulus-response pair, the program displays the best alignment, as well as the unique phonemes in the stimulus and their F1-scores. After all responses are analyzed, the program generates three plots showing the averaged F1-scores (expressed as percentages) for individual phonemes with respect to the classic two dimensional representation of phonological features (1). In these plots, the averaged F1-score is color-coded and assigned at the (*x, y*) coordinates corresponding to the place (*x*) and manner or vowel height (*y*) for each phoneme. A color bar shows the range from 0 to 100 for the F1-score. A fourth plot shows the phonemegram with the relative information transfer for each feature computed as a percentage. Histogram bars are color-coded corresponding to the frequency ranges associated for the features: black was assigned to low frequency for nasality, vowel height, manner and voicing; dark blue assigned to medium frequency for the vowels–contour, vowel place, vowel length; light blue to medium frequency for the consonants–affrication; and white to high frequency for the consonants–sibilance and place.

### Datasets

Two datasets were used. One dataset consisted of test data at sentence and word levels. For the sentences, six volunteers with hearing loss recorded their responses to stimuli. In 2017, four people with various degrees of hearing loss tested the alpha version of the Speech Banana iPad app for auditory training (10); testing was approved by JHU Homewood Institutional Research Board Protocol HIRB00001670. Specifically, the volunteers provided their responses to different sets of 30 sentences recorded in Clear Speech (44) by male and female American English speakers, extracted as WAV audio files from the app which is based on an auditory training book (45). At the same time, two clinical audiologists who also use cochlear implants donated their responses to 19 sentences from AzBio lists #1 and #6 (8) with stimuli presented at 60 dB SPL with 12-talker babble at 50 dB SPL. For the words, stimulus-response pairs were obtained from three sources: a) MSTB [page 12 in (9)], b) List 1 of PBK-50 (2, 46) with the “kints” response to “pinch” observed in a YouTube video clip (https://www.youtube.com/watch?v=GPRwA9BG-m4), and c) erroneous responses to AB word lists (3) by several adults with hearing loss [Table 1-1 in (47)] including the “she's” response to “cheese”. The other dataset consisted of stimulus-response pairs of 31 participants (age range: 22–79 years), each listening to 16 lists of 25 Basic English Lexicon (BEL) sentences (48) at four different SNRs (0, 5, 10, quiet) obtained from speech perception experiments (49, 50); these lists are akin to and more extensive than the BKB-SIN lists (51). For this dataset, protocols (8804M00507) were approved by the Institutional Review Board of the University of Minnesota, and all participants provided written informed consent prior to participating.

### Validation

The large dataset of 12,400 actual stimulus-response pairs from 31 participants listening to 400 sentences is used to validate the program. A set of 12,400 random pairs is created by randomizing the responses such that none of the actual pairs are replicated. Following similar approach (15, 17), three computations are performed. First is a frequency histogram of sentences with the number of correct phonemes in the response (indicated by the number of TPs in the calculation of the F1-scores). Second is the entropy or uncertainty for each of the 39 phonemes obtained from the two confusion matrices for the consonants and the vowels used for the phonemegram. Similar to above, the entropy is calculated as $-\overset{40}{\underset{k=1}{\sum }}p_{k}log_{2}\;p_{k}$ where *k* sums over all the response phonemes as well as unclassified ones due to empty responses (40, 42). Third is the information transfer for the same ten phonological features used in the phonemegram.

## Results

**Figure 6:**
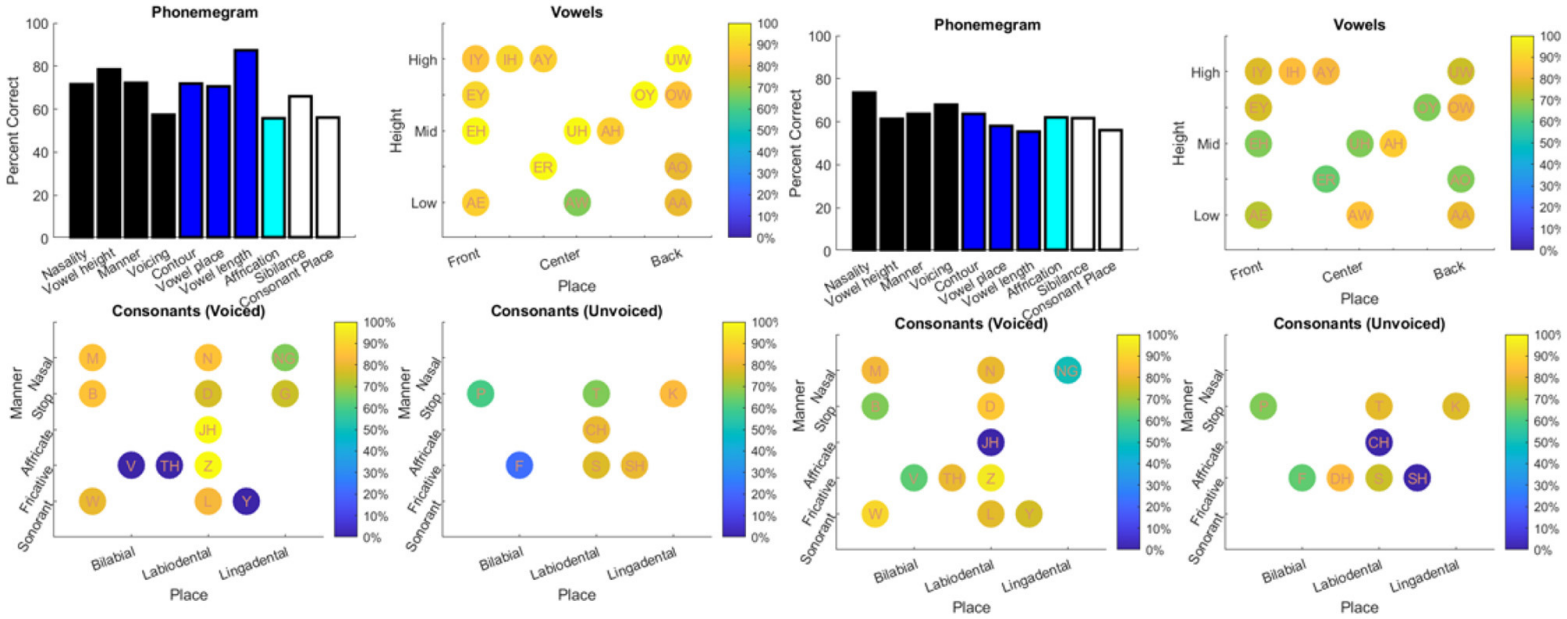
Visual representation of the two typical examples of scoring in the clinic shown in Figure 1. The results from the CNC word list and AzBio List #6 are shown on the left and right respectively. See Table 3 for details.

**Figure 7:**
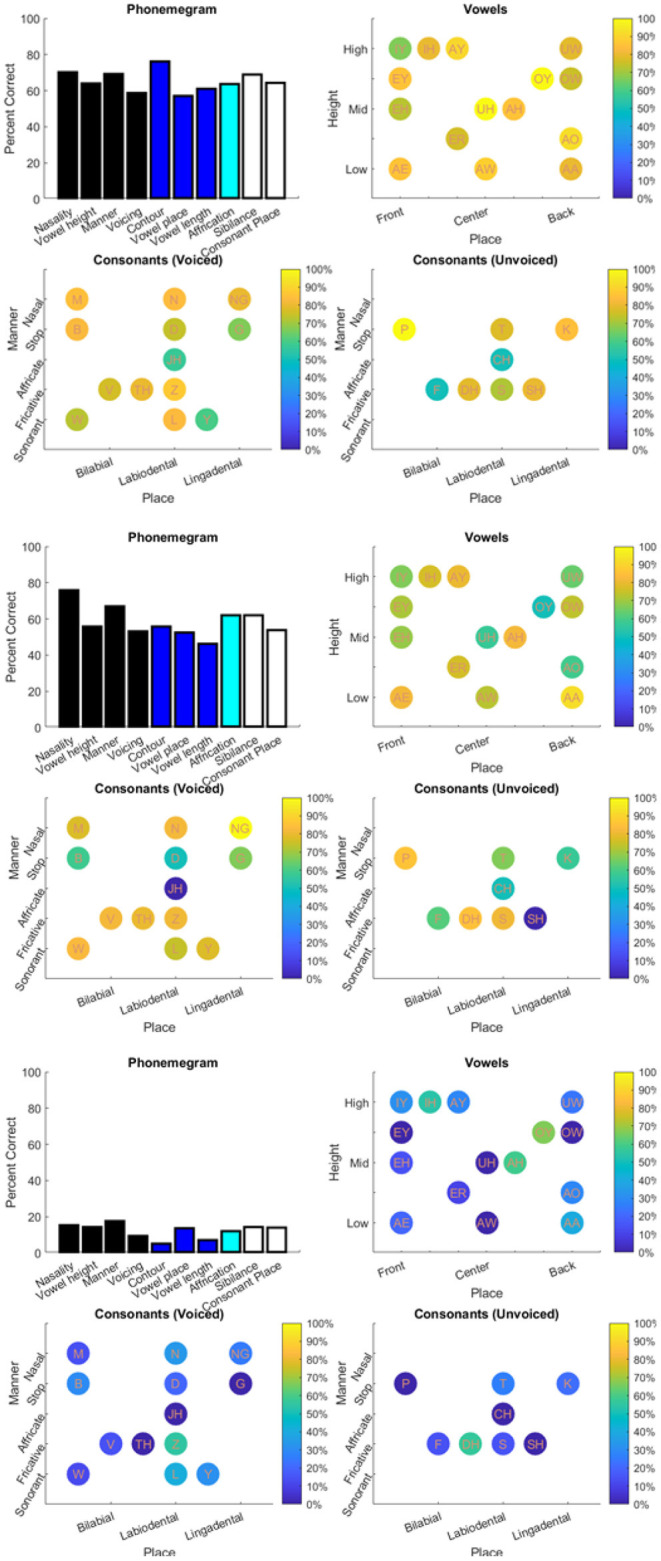
Results for V1 with both cochlear implant (CI) and hearing aid (HA) (**top**), CI only (**middle**) and HA only (**bottom**) in response to a set of 30 sentences extracted from Speech Banana auditory training app. See Table 3 for details.

**Figure 8:**
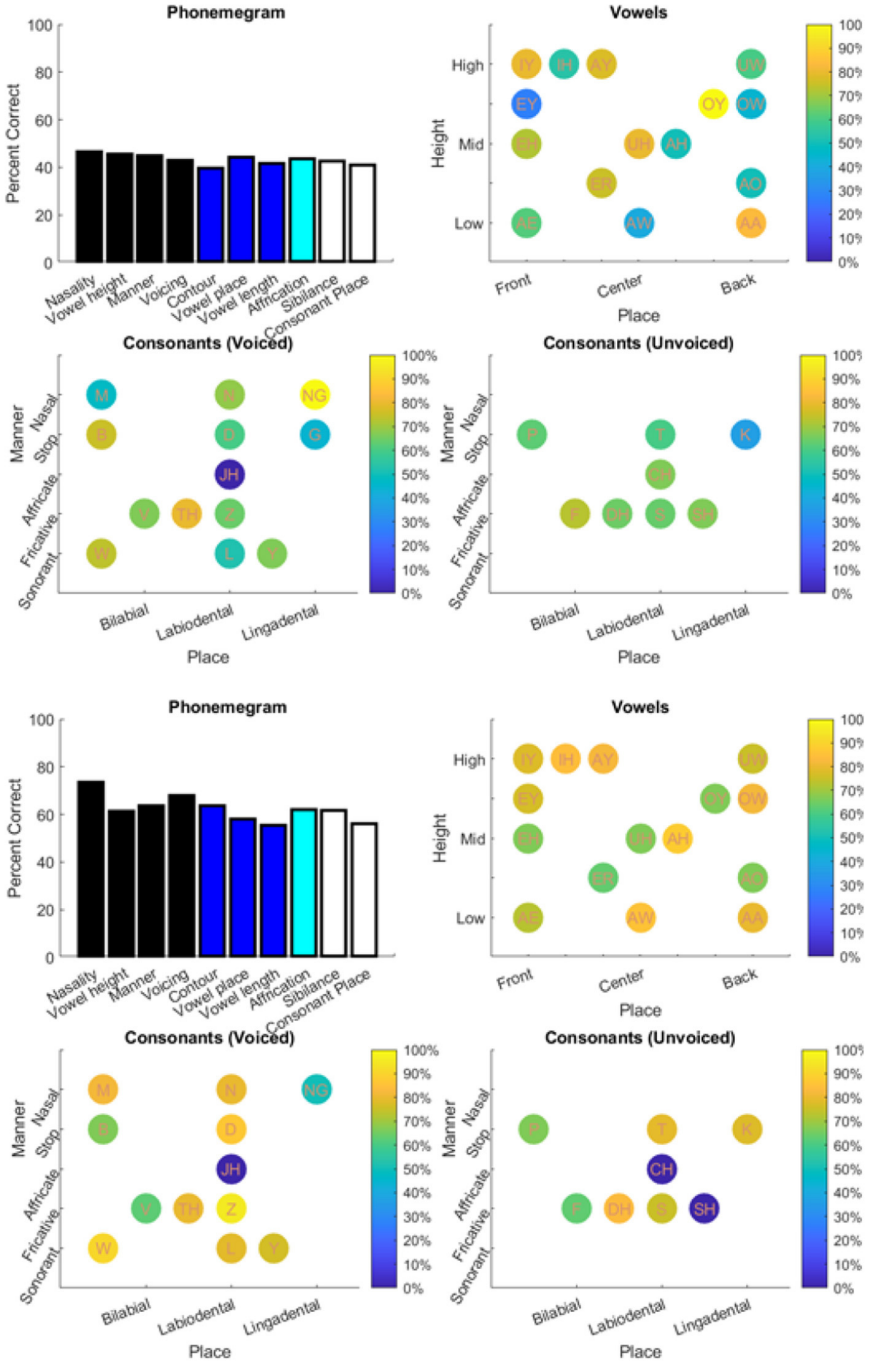
Results for V2 without in the canal HA (**top**) and V3 (**bottom**) with HA responding to different sets of 30 sentences extracted from Speech Banana auditory training app. See Table 3 for details.

**Figure 9:**
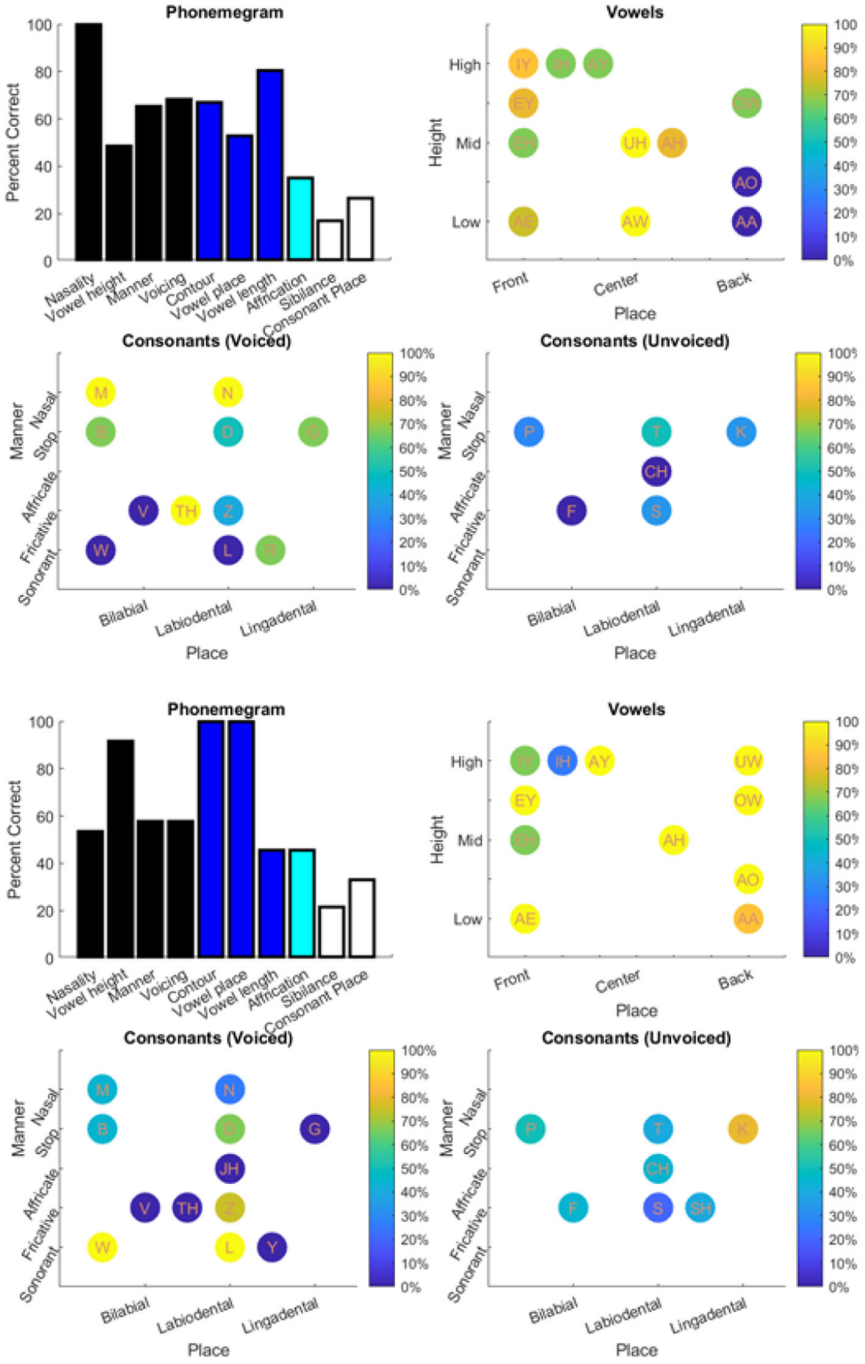
Results for output from two different word tests: PBK-50 (**top**) and AB (**bottom**). See Table 3 for details.

**Figure 10:**
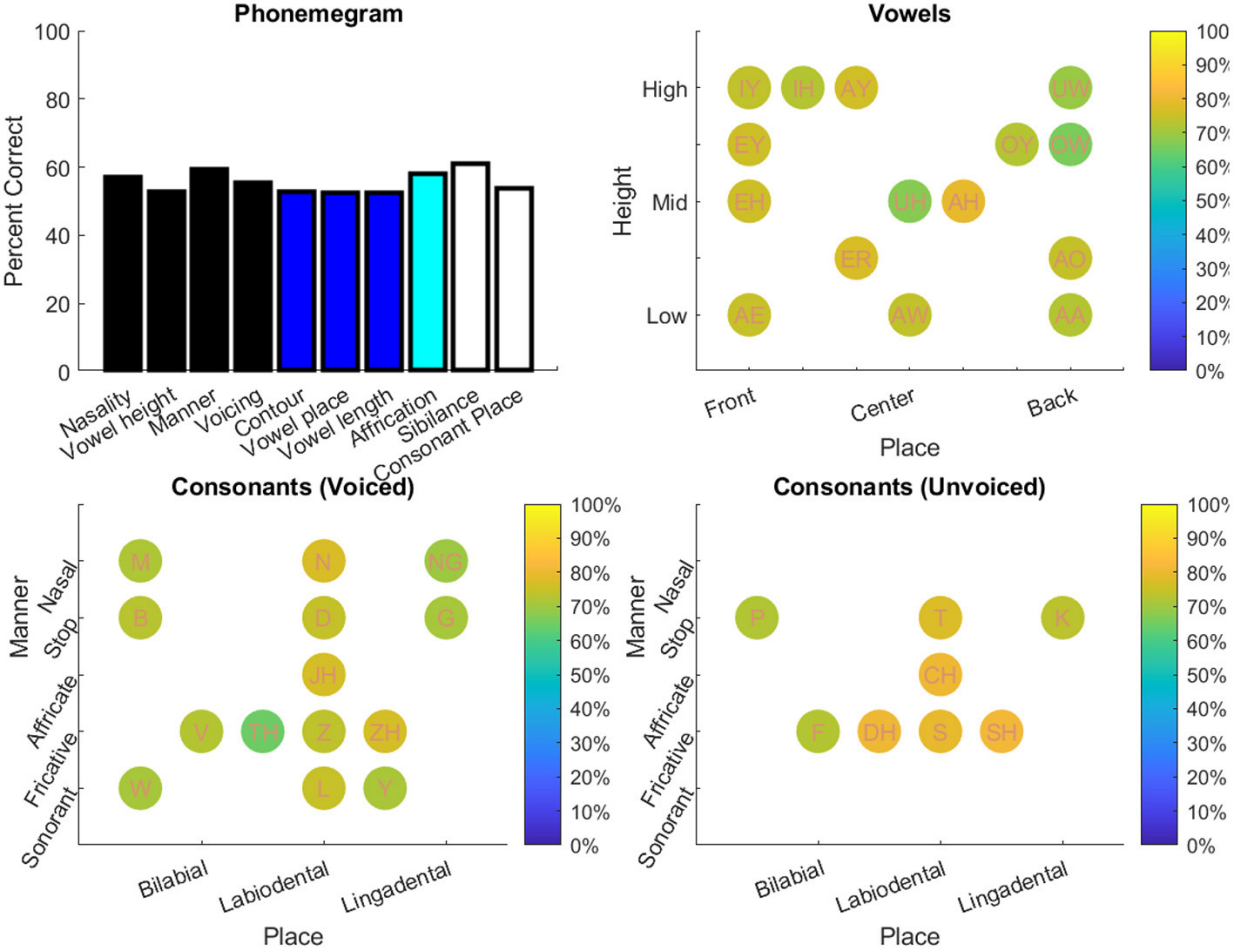
Analysis of the stimulus-response pairs pooled from the O'Neill et al. (50) study of 31 participants with cochlear implants listening to 16 lists of 25 sentences as spoken by four speakers at four different SNR levels. Analysis from the individual participants is shown in Supplementary Figures 2–4. See Table 3 for details.

**Figure 11:**
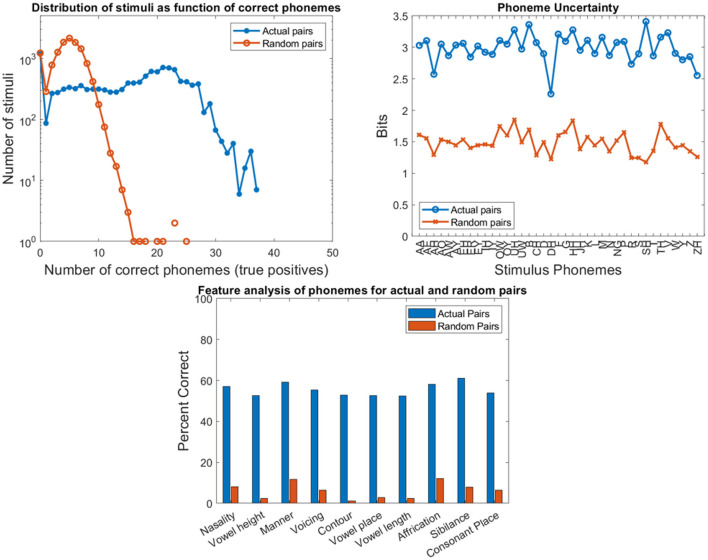
Results of program validation by comparing actual and random 12,400 stimulus-response pairs from the O'Neill et al. (50) study of 31 participants with cochlear implants listening to 16 lists of 25 sentences as spoken by four speakers at four different SNR levels. Top left compares the frequency histograms of stimuli with the number of correct phonemes in the response. Top right compares the entropy or uncertainty for the phonemes. Bottom compares the relative information transfer for the ten phonological features used in the phonemegram.

The results from the two datasets are shown in Table 3, Figures 6–**11**, and Supplementary Figures 1–4. Figure 6 provides the desired visual representation of results in Figure 1 from the two examples from the CNC word list (from the MSTB manual) and AzBio List #6 (by one of the two clinical audiologists with a cochlear implant). Figure 7 shows results for one person with profound congenital bilateral hearing loss (V1), aided bimodally with a cochlear implant and a hearing aid (**top**), unilaterally with just the cochlear implant (**middle**), and unilaterally with just the hearing aid (**bottom**). Figure 8 shows results for one person with severe hearing loss (V2) without using an in the canal hearing aid (**top**) and one person with partial but progressive hearing loss (V3), aided with bilateral hearing aids since childhood (**bottom**). Figure 8 should be compared with Supplementary Figure 1 showing near perfect results from V2 aided with the in the canal hearing aid (**top**), one person with severe progressive hearing loss (V4, **middle**) who has been using bilateral hearing aids for a few years and the other clinical audiologist (V5, **bottom**). Figure 9 shows the results from the two other word lists. Table 3 reports the number of total and correct sentences, words and phonemes for the test and validation datasets, with the last column indicating that the program is able to give comprehensive results in real time; note that the one case of manual intervention, such as entering the phonemes for nonsense responses, resulted in a slightly longer run time. Limiting the analysis to only incorrect stimulus-response pairs did not drastically alter the visualization of phoneme accuracy. Of the 361 stimulus-response pairs used for Figures 6–9, there were just two instances of double alignments. Figure 10 visualizes the pooled results of the responses from 31 participants with cochlear implants listening to lists of BEL sentences as spoken by different talkers at different SNR levels; the runtimes for the individual participants shown in Supplementary Figures S2–S4 ranged from 9.3 to 22.7 secs. Figure 11 shows the validation results by comparing 12,400 actual and random stimulus-response pairs in three different ways. There were just 45 instances of double alignments from the actual pairs. MATLAB scripts including the stimulus-response pairs used to generate these figures (except the validation data) are available from https://github.com/SpeechBanana/SpeechPerceptionTest-PhonemeAnalysis.

## Discussion

In this pilot study, an automated program for visualizing phoneme accuracy in speech perception tests has been developed and implemented. Two key features are the use of a digital speech pronouncing dictionary for automated derivation of the phonemes from stimuli and responses, and the modification of the Levenshtein minimum edit distance via dynamic programming for automated alignment of phonemes. Traditionally, speech pronouncing dictionaries have been used in speech recognition research for purposes such as aligning phonemes in speech-to-text translation (38). Here, the open source CMUDict is used for aligning phonemes in text-to-text comparison. The program is able to parse results (Figure 1) from standard speech tests at the phoneme level (Figure 6) in a robust, efficient, flexible and fast manner.

Several observations can be made. First, there is a benefit from amplification which, however was not an aim of this work. Second, while the averaged F1-scores are informative overall, the phonemegram analysis of the sentences appear to provide less information than that for the word tests which, could be attributed to significant top-down or contextual processing when presented with sentences. Third, there is potentially more information provided by the analysis of phonemes than just the number of correct sentences, words or even phonemes. Here accuracy is viewed in two different ways. The first shows the consequences of inserting, deleting and substituting phonemes and the second shows the perception of the phonological features. Such information about phonemes could help guide auditory training either in the clinic or at home.

A few things can be observed from the validation experiments. First, the likelihood of having five or more exactly matched phonemes for a randomized pair is low (~44%) compared with that for an actual pair (~83%). Second, actual responses can be distinguished from the randomly assigned ones. Third, there is higher uncertainty in response phonemes from random pairs (with a difference of about 1–1.5 bits across all phonemes). Fourth, very little information for the features can be discerned from the randomized pairs. Fifth, the tail of the distributions for actual pairs is higher due to better speech comprehension with cochlear implants even across different SNR levels while the tail for random pairs is influenced by a combination of duplicated pairs and mismatches of just a few words. These observations suggest that the program is robust to the nature of the stimulus-response pairs.

Although automated alignment of phonemes have been used for evaluation of speech recognition systems (52, 53), this study is not the first reported use of automated alignment of phonemes to study speech comprehension by people with hearing loss. The earlier work of Bernstein and colleagues (15–18) mainly focused on lipreading i.e., comprehension via audiovisual stimuli for people with normal hearing and hearing loss and only recently has this focus moved to listening to speech in noise by people with normal hearing (22). Alignment of phonemes via dynamic programming was also used by Ghio and colleagues (19–21) to develop intelligibility metrics for atypical speech. Therefore, it is helpful to discuss similarities and differences between these approaches in four areas: pronunciation dictionary, costs, alignment and metrics.

CMUDict is open-source and has more than 134,000 words, which is an order of magnitude larger than 35,000 words in PhLex used by Seitz, Bernstein, Auer Jr, and MacEachern (54). Words not in CMUDict were manually parsed and entered in the CMUDict website to yield the phonemic string while a rule-based transcription system was used by Bernstein et al. (22). Ghio et al. (20) used a French based pronunciation dictionary (55).

The costs in Table 2 are essentially *ad hoc*, having evolved from the open-source scLite software used for the Levenshtein algorithm. It is worth noting that Bernstein (15) and Bernstein et al. (17) initially used *ad hoc* costs which were fixed with perceptually based costs. Costs for vowel-vowel, consonant-consonant and same consonant manner alignments were modified based on having similar phonological features. In fact, a similar approach has been adopted by Ghio and colleagues (19–21) and Kondrak (56, 57) who used Hamming distance matrices for vowels and consonants based on deviations from features for the costs used by dynamic programming for analyzing atypical speech and different languages, respectively; Ruiz and Federico (38) used a similar approach with constraints for vowels and consonants in analyzing speech translation. The Supplementary Data shows that the phoneme pairs deemed to be similar can actually be derived by thresholding the distance matrix for the vowels and the stratified distance matrices for the consonants. It should then be possible to make formal use of these distance matrices. While voicing was not explicitly used in setting costs, it was actually used to determine the costs for consonant-consonant substitution pairs. As described in the Supplementary Data, the sibilant consonants were grouped and then non-sibilant consonants were stratified based on first manner, then place and voicing. In contrast, Bernstein et al. (22) perceptually computes costs based on the Euclidean distance between two phonemes derived from multidimensional scaling of confusion matrices for consonants and vowels from people with normal hearing. Since further work should compare the feature- and perceptual- based approaches, this work should be considered as a pilot study.

The use of modified costs in MED operations to align the phonemes in Figure 2 should be contrasted with that in Figure 1 in Bernstein (15). Usually, MED operations yield multiple alignments (Figure 4); see also Bernstein et al. (17) and Figure 2 in Bernstein (15). In this work as well as the recent work by Bernstein et al. (22) and Ghio et al. (20), single alignments are achieved in virtually all cases which may be attributed to the use of costs derived from the distance matrices. About 0.6% of the stimulus-response pairs in both test (*n* = 2) and validation (*n* = 45) datasets yielded multiple—actually double—alignments. In the rare case of double alignments, the user is given the manual option of choosing the best one; by default, the program selects the first of the two alignments. It is remarkable that only double alignments occurred; in fact, more than two alignments occurred when a lower cost of two instead of five for consonant-vowel substitution was used. Inspection of the 45 stimulus-response pairs from the validation dataset that yielded double alignments suggests that these arise depending on the type of the response. The response may be nearly complete such that the alignment cannot decide between two similar phonemes, or a purely random guess, or a combination of correct and random words. This is actually borne by instances of double alignments from 2.5% (*n* = 305) of the randomized stimulus-response pairs from the validation dataset. Avoidance or significant reduction of multiple alignments using feature-based costs were also observed in a comparative study of Dutch dialects (58). As this work is a pilot study, further work should explore differences accrued from feature-based *ad hoc* and perceptual-based costs. These differences might be reflected by comparing the alignments for 12 stimulus-response pairs listed in Table 1 of Bernstein et al. (22) with those produced by the program in the Supplementary Data. There may be problems with sparse responses such as misaligning one response phoneme in a correct word with the stimulus phoneme in a different (as in a preceding) word, ironically without loss of accuracy so future work should incorporate costs for boundary detection (38). These problems are likely not to occur with word lists or nearly complete sentences which may be more helpful in pinpointing areas of weaknesses for auditory training. Others have used MED for aligning phonetic transcriptions of words based on phonological features (59) and fuzzy string matching with a novel metric for sentences (60), both of which are available as open source. Future work should also explore using costs from confusion matrices from people with normal hearing listening to sentences as opposed to words.

In this work, two sets of commonly used metrics are used. One is the F1-score which is a function of true positives, true negatives and false positives for each phoneme and visualized with respect to manner, place and voicing for consonants and height and place for vowels. The other is the relative informationtransfer or entropy for each of the 10 phonological features used to construct the phonemegram. In contrast, the recent work of Bernstein et al. (22) proposed mining three metrics to analyze listening by people with normal hearing to speech in noise. These were (i) phoneme substitution dissimilarity, which measures the perceptual distance between separate stimulus phonemes and all incorrect phonemes in the response, (ii) number of words correct, and (iii) number of insertions. The former is obtained from dividing the sum of the phoneme-to-phoneme costs for incorrect substitutions by the number of substitutions. The latter is obtained by the count of the number of phonemes that could not be aligned as substitutions. In contrast, the program did not save these types of data except for the number of true positives needed for the validation study (Figure 11, top left). It is argued that due to different manipulations of intrinsic data the two different set of metrics are probably related in some way or other. Furthermore, in analyzing people with speech disorders, Ghio et al. (20) used the distance between the expected and actual sequence. As this is a pilot study, future work would be necessary to uncover and explore these relationships particularly in a comparison i.e., statistical study.

Care must be taken to interpret the accuracy for phonological features. Take, for example, analysis of several people with hearing loss in the bottom panel of Figure 9. The near-perfect scores for vowel height, contour, and vowel place may seem inaccurate but the phonological analysis of the vowels show an inability to identify IY and IH. Since these vowels are grouped for the vowel height, contour, and vowel place features, accuracy for identifying phonemes with these features remains at 100%. In other words, even though there may have been confusion between IY with IH, since both are identical with respect to their categorization within the vowel height, contour, and vowel place feature groups, the responses showed the ability to detect those features at a high rate. Similarly, for the PBK-50 test (Figure 9, **top**), since the non-nasal consonants are still categorized as the same the nasality feature is recorded perfectly.

The availability of datasets from recently published experiments provided an opportunity to assess the potential use of phoneme alignment in these experiments. For example, O'Neill et al. (49) recorded the BEL sentences using four different talkers, as well as developed and recorded 20 lists of nonsense sentences derived from the BEL corpus. These stimuli were used in speech perception experiments involving people with normal hearing and hearing loss (49, 50). The visualization of phoneme accuracy from Supplementary Figures S2–S4 for one experiment (50) provides potentially more information than the reported percentage of correctly identified keywords in sentences.

By construction, the phonemegram offers a different perspective of speech comprehension based on phonological features of the phonemes, specifically information transfer of features with respect to a frequency range, as used in the Infogram for hearing aid fitting in tele-audiology (23–25). Information transfer analysis has also been adopted (32, 39) who compared low frequency phonemes (diphthongs, semivowels, and nasals) to high frequency phonemes (sibilants, fricatives, bursts, and plosives). The frequency aspect of the phonemegram may be complemented by the averaged F1-scores for the vowels based on the inverse relationship between place from back to front (manner from high to low) and the 1st (2nd) formant (1). Further, the phonemegram can compensate for the absence of variance for the F1-scores since it records just the information transfer for a feature. It is important to provide enough repetitions for each phoneme, otherwise the transmitted information estimate becomes highly erratic and overestimates the stimulus information on average (43, 61). Accumulating responses over time is one way to overcome bias and error which might be useful in mobile apps for auditory training (10). In this case, it may be necessary to use bootstrapping to generate confidence intervals (62). Furthermore, since non-symmetric confusion matrices have been considered in analysis of speech perception by people with hearing loss (40, 42), it is reasonable to consider non-classified phonemes accruing from empty responses. Further work could consider a more appropriate alternative visualization by generating 3D plots of F1-scores for each phoneme with respect to the first three formants.

A challenge for testing the program was obtaining examples of stimulus-response pairs from people with hearing loss. Fortunately, the program was developed at the same time as the development of the Speech Banana mobile app for auditory training which allowed for testers to provide valuable data. In this era of digital hearing health, there is a great need for raw data such as stimulus-response pairs from scientific studies to be made available publicly in the same way as human neuroimaging data are now being made available for the scientific community (63, 64). The use of the data from recently published speech perception experiments is a step in that direction.

The program has several other advantages. First, though currently implemented in Matlab, the program can be implemented in Python, Javascript or even R. Second, it could be self-administered or used in telepractice by people with hearing loss, who are learning to hear with a new hearing aid or cochlear implant. Results are saved over time for feedback with the speech language pathologist or audiologist. Third, the program could be integrated with inputs from NU-6, CUNY, Az-Bio, HINT or BKB for real-time quantification in the clinic; further, the program could be integrated with more challenging tests such as Az-TIMIT (65), STARR (66) and PRESTO (67). Fourth, as implied by the Infogram, the phonemegram may offer audiologists a frame of reference for the ability of the person with hearing loss to perceive speech at different frequencies. Fifth, the visualization of phonemic accuracy may offer speech language pathologists a perspective of how the person with hearing loss processes different phonemes, in order to develop a targeted auditory training program. Sixth, the program can be used for educational purposes. For instance, it was used in the past few years for assessing responses by biomedical engineering undergraduates at Johns Hopkins University doing the Speech Perception module of the Neuroengineering Lab in which they listened to sentences in simulations of different types of hearing loss, number of channels in a cochlear implant, and frequency offsets in a cochlear implant.

There are also several disadvantages. Many people with hearing loss use top-down processing such as using contextual information to fill in words misheard in sentences (50, 68–71), so accuracy of responses to sentences may be overestimated. In fact, this may explain the small differences between the information transfer values for the features with the sentences in the test cases. As alluded above, word lists may be more practical in the clinic (Figures 6, 9). Differences in stresses and emotion may influence perception (72) and therefore, might require using lexical stress information, if available, from the pronouncing dictionary. Finally, the program may not be suitable for people with very poor speech comprehension as they are more than likely to make random or very sparse guesses that may then confound phoneme alignment. For example, the last stimulus-response pair in Figure 5 yielded alignment that was erratic with respect to the first part of the stimulus due to the volunteer having greater difficulty hearing with just the hearing aid instead of bimodal hearing. Incidentally, this is a good example of a person with hearing loss finding it difficult to process the early part of a stimulus compared with the rest of the stimulus (71).

Future work includes feasibility for clinical usage, user-friendly implementation for mobile auditory training apps such as Speech Banana (10), and exploring alternative approaches such as multidimensional scaling for features (29, 30) as opposed to prescribed ones, other features (69) and other metrics (6, 16, 73).

## Data Availability Statement

The raw data supporting the conclusions of this article will be made available by the authors, without undue reservation.

## Ethics Statement

The first dataset consisted of responses to sentences by volunteers testing the Speech Banana app. For this dataset, protocol (HIRB00001670) was reviewed and approved by JHU Homewood Institutional Research Board. The other dataset consisted of stimulus-response pairs of 31 participants. For this dataset, protocol (8804M00507) was approved by the Institutional Review Board of the University of Minnesota, and in both cases, all participants provided written informed consent prior to participating.

## Author Contributions

Concept was developed by JR and DT. Algorithmic development was by LW, S-HB, and ES. Testing and manuscript was written by JR, LW, S-HB, and EO'N. All authors contributed to the article and approved the submitted version.

## Funding

EO'N was supported by NIH grant R01 DC012262 awarded to Professor Andrew Oxenham of the University of Minnesota.

## Conflict of Interest

The authors declare that the research was conducted in the absence of any commercial or financial relationships that could be construed as a potential conflict of interest.

## Publisher's Note

All claims expressed in this article are solely those of the authors and do not necessarily represent those of their affiliated organizations, or those of the publisher, the editors and the reviewers. Any product that may be evaluated in this article, or claim that may be made by its manufacturer, is not guaranteed or endorsed by the publisher.
