# Supplementary material for: Visualization of Speech Perception Analysis via Phoneme Alignment: A Pilot Study
Source: Front Neurol. 2022 Jan 11;12:724800. doi: 10.3389/fneur.2021.724800 (PMC8787339; doi:10.3389/fneur.2021.724800)
Supplement: Supplementary file 1 [file Data_Sheet_1.pdf]

SUPPLEMENTARY DATA

Visualization of Speech Perception Analysis via Phoneme Alignment: a pilot study

J. Tilak Ratnanather[1], Lydia C. Wang[1], Seung-Ho Bae[1], Erin R. O'Neill[2], Elad Sagi[3], Daniel J. Tward[1,4]

[1]Center for Imaging Science and Institute for Computational Medicine, Department of Biomedical Engineering, Johns Hopkins University, Baltimore, MD 21218
[2]Center for Applied and Translational Sensory Sciences, University of Minnesota, Minneapolis, MN 55455
[3]Department of Otolaryngology, New York University School of Medicine, New York, NY 10016
[4]Departments of Computational Medicine and Neurology, University of California Los Angeles, Los Angeles, CA 90095

Pairs of vowels and consonants with similar phonological features uses for substitution

This section describes the derivation of pairs of phonemes with similar phonological features. Table 1 is used to build a Hamming distance matrix as follows. Given M phonemes and N features. $F \in \mathbb{R}^{M\times N}$ is a feature matrix with positive integer values, i.e., $F_{ij} \in \{0,1,2,..,F_{j_{\max}}\}$ where $j_{\max}$is the number of subtypes for the j-th feature. Construct $D \in \mathbb{R}^{M\times M}$such that $D_{ij} = \sum_{k=1}^{N} \mathbb{I}\left(F_{ik} \neq F_{jk}\right)$ where $\mathbb{I}$ is the indicator function and threshold $D_{ij} > TH$ to yield pairs of phonemes that have more than TH features in common.

Vowel-vowel pairs: The figure below shows the initial and thresholded (with $TH = 2$) distance matrices for the 15 vowels with 3 or more common features.

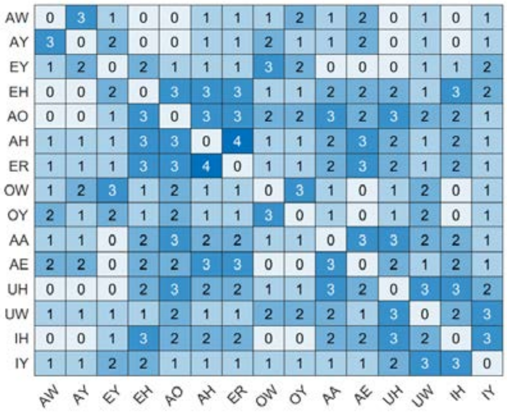

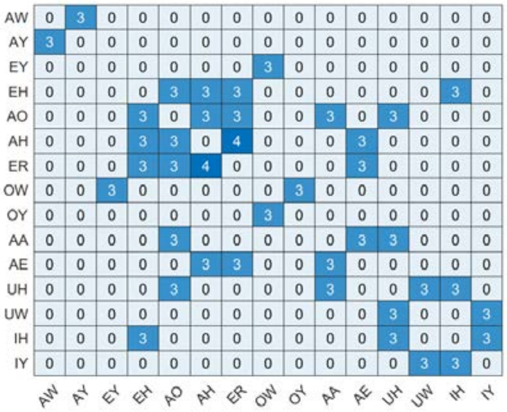

The following vowel-vowel pairs with more than two common features are:

1) AA↔AO, AE, UH
2) AE↔AH, ER, AA
3) AH↔EH, AO, ER, AE
4) AO↔EH, AH, ER, AA, UH
5) AW↔AY
6) AY↔AW
7) EH↔AO, AH, ER, IH
8) ER↔EH, AO, AH, AE
9) EY↔OW
10) IH↔EH, UH, IY
11) IY↔UW, IH
12) OW↔EY, OY
13) OY↔OW
14) UH↔AO, AA, UW, IH
15) UW↔UH, IY

Consonant-Consonant pairs: With 24 consonants and many features and subtypes, Table 1 was used to stratify the consonants based on sibilance. First, the sibilant consonants were grouped. Then the non-sibilant consonants were stratified based on first manner, then place and voicing. This yielded the following:

Sibilants
same: S↔SH, CH↔JH, Z↔ZH;
manner, affrication, place: S↔Z, ZH, SH↔Z, ZH, CH↔JH
voice, affrication, place: SH↔CH, ZH↔JH
Non-sibilants

Stops – P↔B, T↔D, G↔K
Nasals (voice or unvoiced): M↔P, B, N↔T,D, NG↔G,K
Voice: T↔K, D↔G, G↔D, K↔T
Fricatives: F↔DH, TH↔V
Fricatives (voice or unvoiced): F, DH↔TH,V
Glides: R↔L↔Y

This stratification excludes HH and W which are classified as aspirate and semi-vowels, and is reflected by the following sequence of three distance matrices corresponding to the original order of the consonants (top), rearranged order based on the above stratification (lower left) and finally thresholded (lower right) as having 5 or more common features (with $TH = 4$).

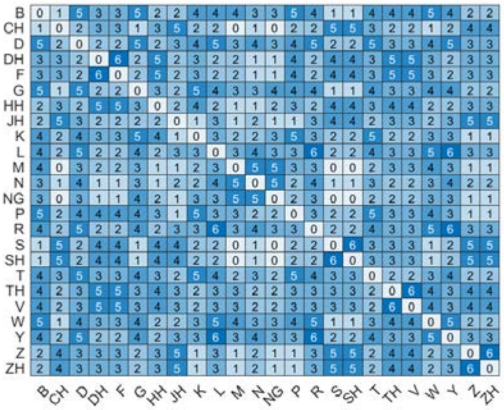

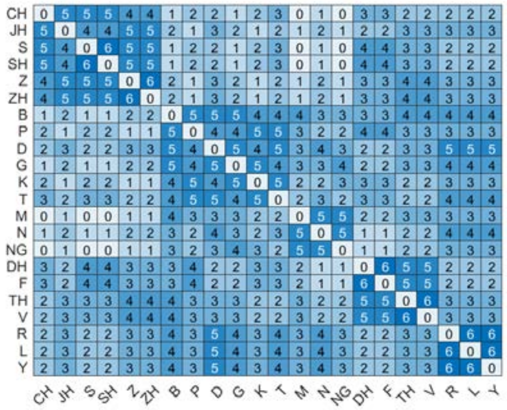

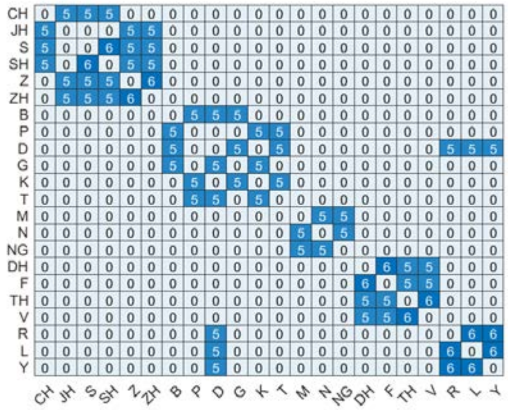

<u>Same consonant manner pairs:</u> As it is necessary to find other consonants confused with HH and W, consider those with the same manner values of 0, 2 and 3, i.e., exclude nasal and affricative pairs with the same manner (CH & JH with manner 4 and M, N & NG with manner 1 respectively). Subsequent analysis of 7575 non-duplicated stimulus-response pairs used for validation showed that some consonants in the same manner substitution set were not used; these are indicated with a strikethrough.

1. B D, G, K, ~~P~~, T Manner 0
2. D B, ~~G~~, K, P, ~~T~~ Manner 0
3. G B, ~~D~~, ~~K~~, P, T Manner 0
4. T B, ~~D~~, G, ~~K~~, P Manner 0
5. K B, D, ~~G~~, P, ~~T~~ Manner 0
6. P ~~B~~, D, G, K, T Manner 2
7. DH HH, ~~F~~, S, SH, ~~TH~~, V, Z, ZH Manner 2

8.	F	HH, ~~DH~~, S, SH, TH, ~~V~~, Z, ZH	Manner 2
9.	HH	DH, F, S, SH, TH, V, Z, ZH	Manner 2
10.	S	HH, DH, F, ~~SH~~, TH, V, Z, ZH	Manner 2
11.	SH	HH, DH, F, ~~S~~, TH, V, Z, ~~ZH~~	Manner 2
12.	Z	HH, ~~DH~~, F, ~~S~~, SH, TH, V, ~~ZH~~	Manner 2
13.	ZH	HH, DH, F, S, ~~SH~~, TH, V, ~~Z~~	Manner 2
14.	TH	HH, ~~DH~~, F, S, SH, ~~V~~, Z, ~~ZH~~	Manner 2
15.	V	HH, DH, ~~F~~, S, SH, ~~TH~~, Z, ZH	Manner 2
16.	L	~~R~~, W, ~~Y~~	Manner 3
17.	R	~~L~~, W, ~~Y~~	Manner 3
18.	W	L, R, Y	Manner 3
19.	Y	~~L~~, ~~R~~, W	Manner 3

This stratification is reflected by the following sequence of three distance matrices corresponding to the original order of the consonants (as above), rearranged order based on the above stratification (left) and finally thresholded (right) as having 4 or more common features (with $TH = 3$).

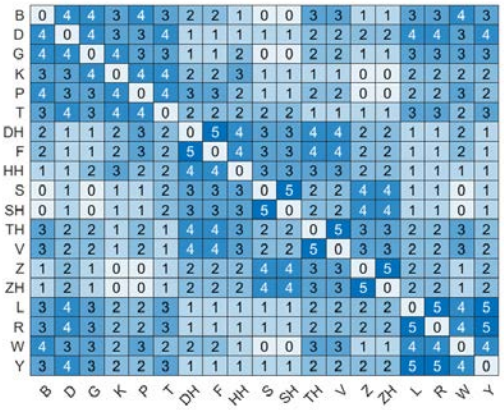

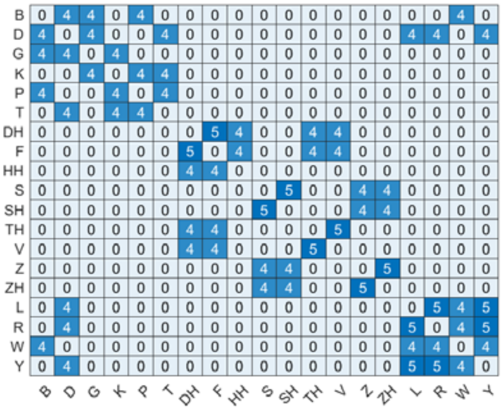

<u>Summary</u>

Consonants with similar manner	Similar consonants	Similar vowels
B, D, G, K, P, T	B, P, M	AA, AO, AE, ER, AH, EH
DH, HH, F, S, SH, TH, V, Z, ZH	CH, JH, SH	EY, OY, OW
L, R, W, Y	D, T, N, M, NG, G, K	UW, UH, IY, IH
	DH, F, TH, V	AW, AY
	L, R, Y	
	S, Z, ZH	

Subsequent analysis of the 7575 non-duplicated stimulus-response pairs used for validation revealed that there were 120305, 136725 and 94158 substitutions for pairs of consonants with same manner, consonants and vowels respectively. For reference, there were 122013 response phonemes, 172034 stimulus phonemes, 20985 out of 45457 correct words and 758 correct responses.

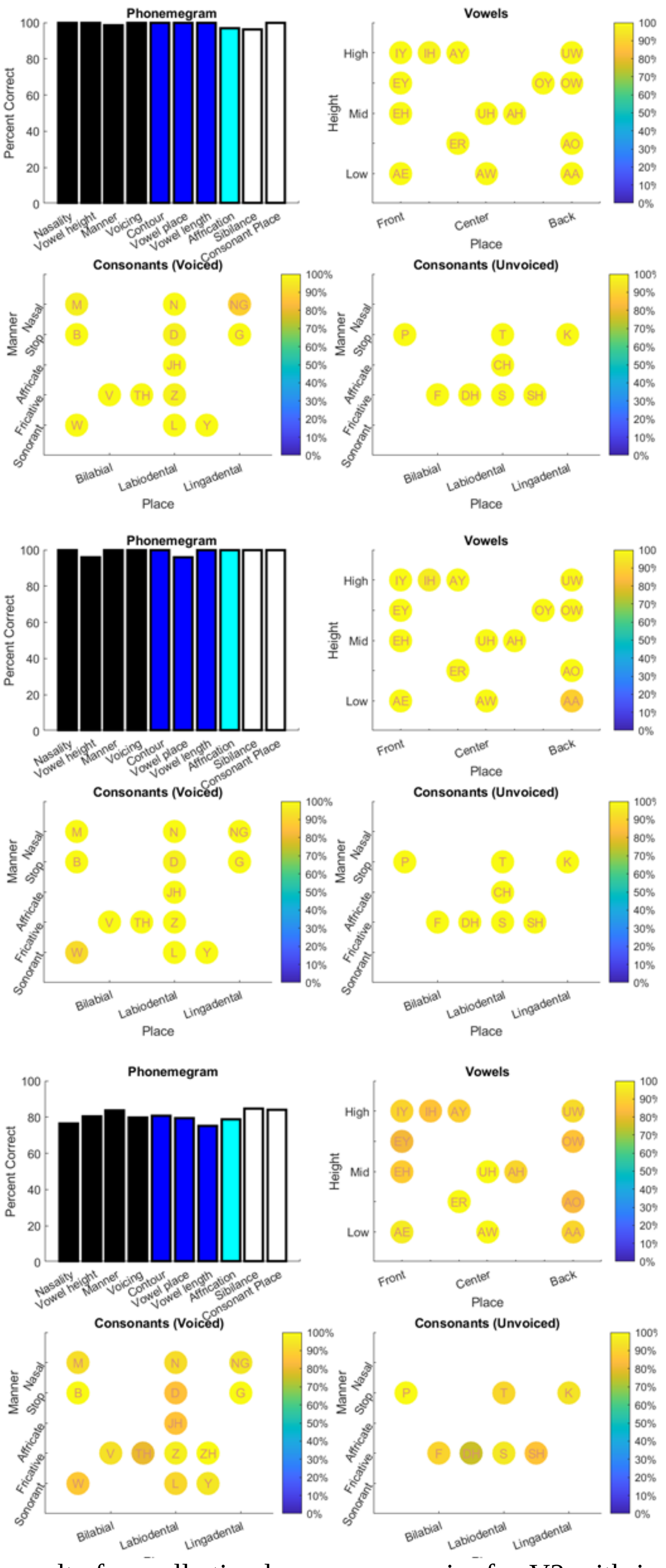

Figure S1: Near perfect results from all stimulus-response pairs for V2 with in the canal HA (top), V4 (middle) with HA responding to different sets of 30 sentences extracted from Speech Banana auditory training app; and V5 (bottom) responding to 19 sentences from the AzBio List #1. See Table 3 for details.

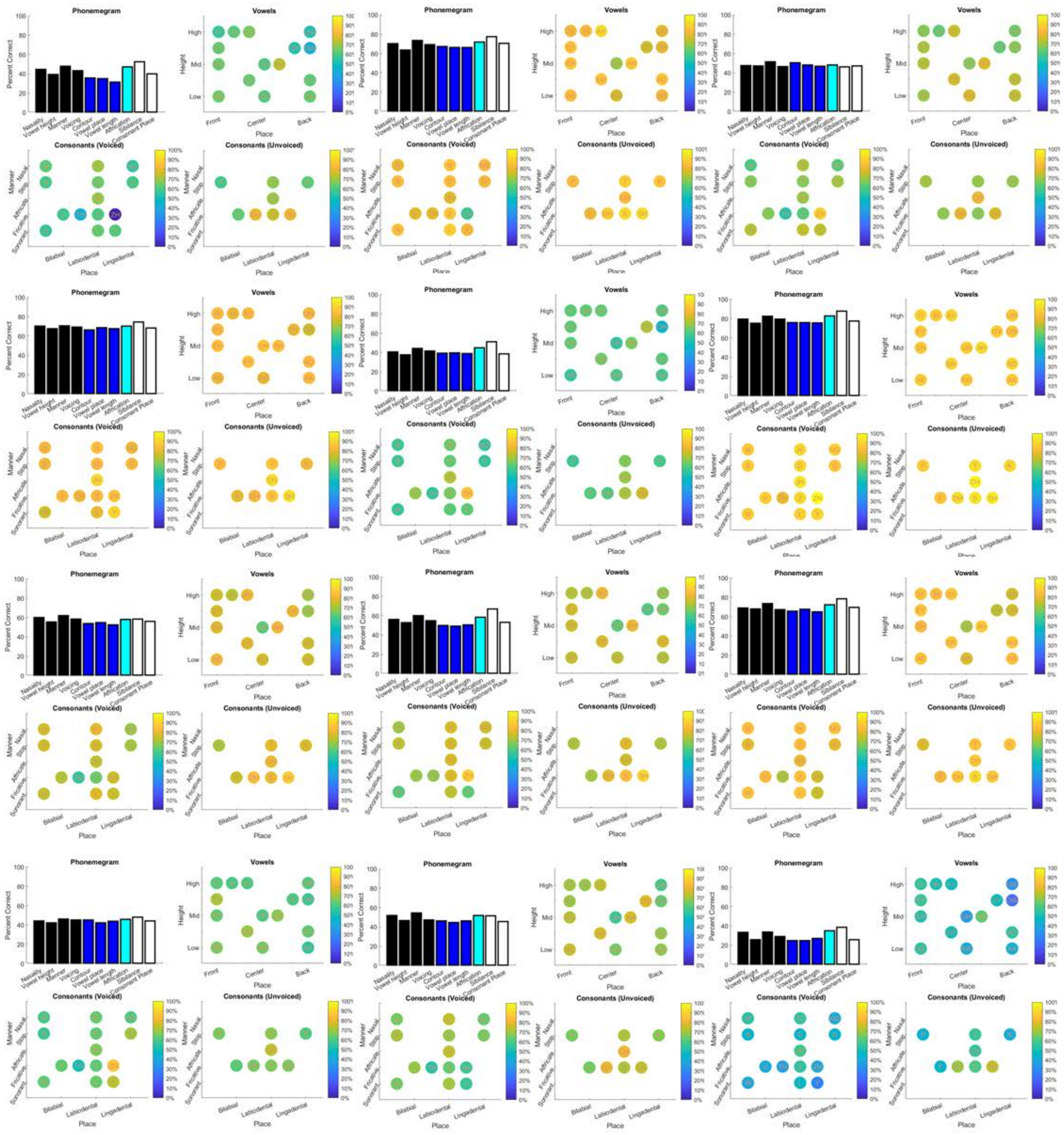

Figure S2: Individual analysis of responses from the O'Neill et al. (2021) study of 31 participants with cochlear implants (#1-#12 shown here from top left to bottom right) to 16 lists of 25 Basic English Lexicon (BEL) sentences for different SNR levels (0, 5, 10, quiet) and different talkers.

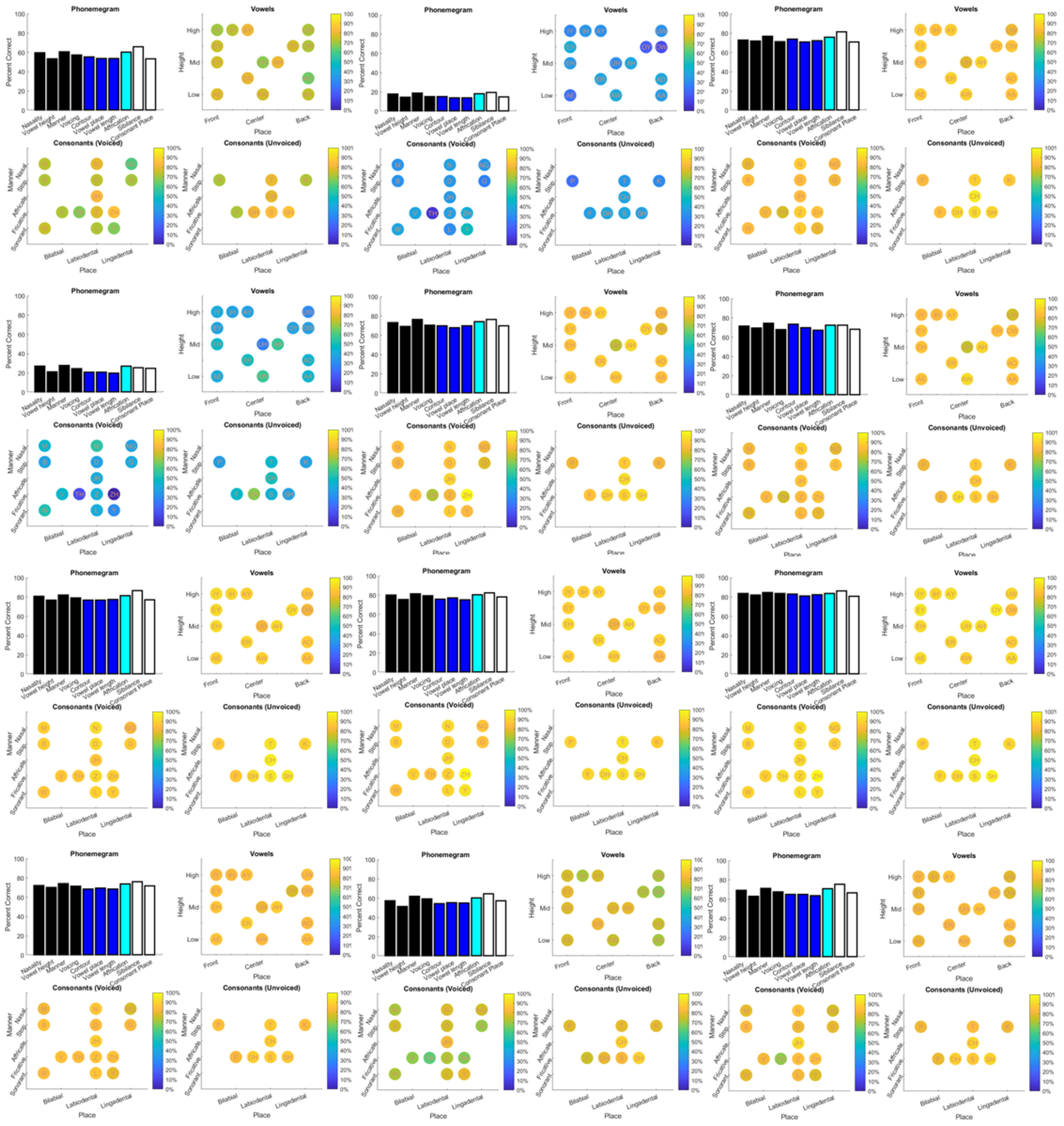

Figure S3: Individual analysis of responses from the O'Neill et al. (2021) study of 31 participants with cochlear implants (#13-#24 shown here from top left to bottom right) to 16 lists of 25 Basic English Lexicon (BEL) sentences for different SNR levels (0, 5, 10, quiet) and different talkers.

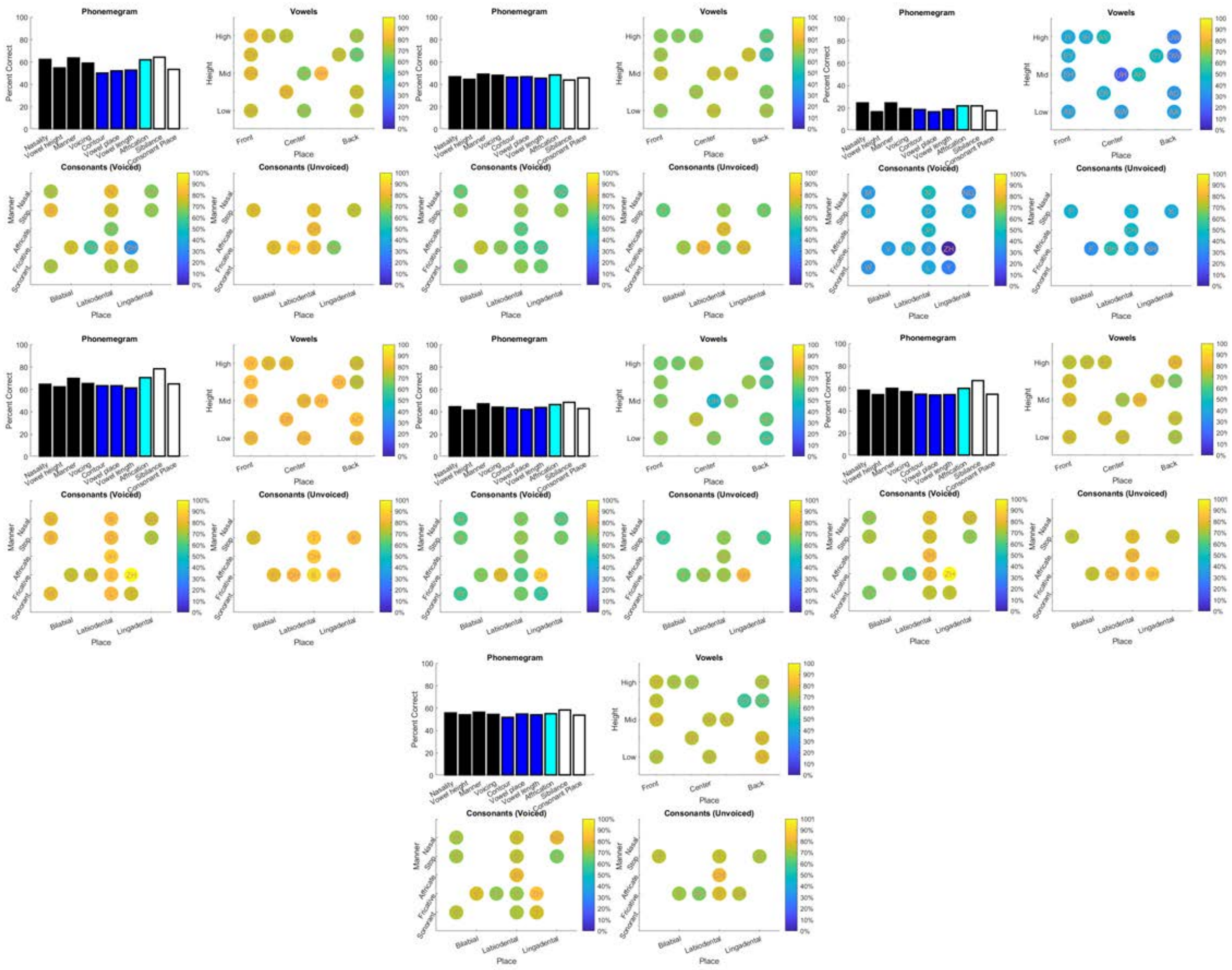

Figure S4: Individual analysis of responses from the O'Neill et al. (2021) study of 31 participants with cochlear implants (#25-#31 shown here from top left to bottom right) to 16 lists of 25 Basic English Lexicon (BEL) sentences for different SNR levels (0, 5, 10, quiet) and different talkers.

Name: Bernstein 2021 Example 1. Stimulus Number: 1 out of 1 stimuli.
Stimulus: The weight of the package was seen on the high scale
Response: The weight of axe was seen Serbia

DH	AH	W	EY	T	AH	V	DH	AH	P	AE	K	AH
DH	AH	W	EY	T	AH	V	_	_	_	AE	K	_

JH	W	AA	Z	S	IY	N	AA	N	DH	AH	HH	AY
S	W	AA	Z	S	IY	N	_	_	S	ER	B	IY

S	K	EY	L
_	_	AH	_

AA	AE	AH	AY	DH	EY	HH	IY	JH	K	L	N	P
66.66	100	50	0	50	66.66	0	66.66	0	66.66	0	66.66	0

S	T	V	W	Z
40	100	100	100	100

~~~~~~~~~~ ~~~~~~~~~~ ~~~~~~~~~~ ~~~~~~~~~~ ~~~~~~~~~~ ~~~~~~~~~~

Name: Bernstein 2021 Example 2. Stimulus Number: 1 out of 1 stimuli.
Stimulus: The weight of the package was seen on the high scale
Response: package tree secure

DH	AH	W	EY	T	AH	V	DH	AH	P	AE	K	AH
_	_	_	_	_	_	_	_	_	P	AE	K	AH

JH	_	W	AA	Z	S	IY	N	AA	N	DH	AH	HH
JH	T	R	IY	_	S	IH	_	_	_	_	_	_

AY	S	K	_	EY	L
_	_	K	Y	UH	R

AA	AE	AH	AY	DH	EY	HH	IY	JH	K	L	N	P
0	100	33.33	0	0	0	0	0	100	100	0	0	100

S	T	V	W	Z
66.66	0	0	0	0

~~~~~~~~~~ ~~~~~~~~~~ ~~~~~~~~~~ ~~~~~~~~~~ ~~~~~~~~~~ ~~~~~~~~~~

Name: Bernstein 2021 Example 3. Stimulus Number: 1 out of 1 stimuli.
Stimulus: The weight of the package was seen on the high scale
Response: just by the ice skater by the

DH	AH	W	EY	T	_	AH	V	DH	AH	P	AE	K
JH	AH	S	_	T	B	AY	_	DH	AH	_	_	_

AH	JH	W	AA	Z	S	_	IY	N	AA	N	_	DH
_	_	_	AY	S	S	K	EY	T	ER	B	AY	DH

AH	HH	AY	S	K	EY	L
AH	_	_	_	_	_	_

AA	AE	AH	AY	DH	EY	HH	IY	JH	K	L	N	P
0	0	75	0	80	0	0	0	0	0	0	0	0

S	T	V	W	Z
40	66.66	0	0	0

~~~~~~~~~~ ~~~~~~~~~~ ~~~~~~~~~~ ~~~~~~~~~~ ~~~~~~~~~~ ~~~~~~~~~~

Name: Bernstein 2021 Example 4. Stimulus Number: 1 out of 1 stimuli.
Stimulus: The weight of the package was seen on the high scale
Response: the weight of the package free and secure

DH	AH	W	EY	T	AH	V	DH	AH	P	AE	K	AH
DH	AH	W	EY	T	AH	V	DH	AH	P	AE	K	AH

JH	_	W	AA	Z	S	IY	N	AA	N	DH	AH	HH
JH	F	R	_	_	_	IY	_	AH	N	D	_	_

AY	S	_	K	_	EY	L
_	S	IH	K	Y	UH	R

AA	AE	AH	AY	DH	EY	HH	IY	JH	K	L	N	P
0	100	80	0	80	66.66	0	100	100	100	0	66.66	100

S	T	V	W	Z
66.66	100	100	66.66	0

~~~~~~~~~~ ~~~~~~~~~~ ~~~~~~~~~~ ~~~~~~~~~~ ~~~~~~~~~~ ~~~~~~~~~~

Name: Bernstein 2021 Example 5. Stimulus Number: 1 out of 1 stimuli.
Stimulus: The square peg will settle in the round hole
Response: The square peg were orifice

DH	AH	S	K	W	EH	R	P	EH	G	W	IH	L
DH	AH	S	K	W	EH	R	P	EH	G	W	ER	_
S	EH	T	AH	L	IH	N	DH	AH	R	AW	N	D
_	_	_	_	_	_	_	_	AO	R	AH	F	_
HH	OW	L										
_	AH	S										

AH	AW	D	DH	EH	G	HH	IH	K	L	N	OW	P
33.33	0	0	66.66	80	100	0	0	100	0	0	0	100
R	S	T	W									
100	50	0	100									

~~~~~~~~~~ ~~~~~~~~~~ ~~~~~~~~~~ ~~~~~~~~~~ ~~~~~~~~~~ ~~~~~~~~~~

Name: Bernstein 2021 Example 6. Stimulus Number: 1 out of 1 stimuli.
Stimulus: The square peg will settle in the round hole
Response: The square pegs were old

DH	AH	S	K	W	EH	R	P	EH	G	_	W	IH
DH	AH	S	K	W	EH	R	P	EH	G	Z	W	ER
L	S	EH	T	AH	L	IH	N	DH	AH	R	AW	N
_	_	_	_	OW	L	_	_	_	_	_	_	_
D	HH	OW	L									
D	_	_	_									

AH	AW	D	DH	EH	G	HH	IH	K	L	N	OW	P
50	0	100	66.66	80	100	0	0	100	50	0	0	100
R	S	T	W									
66.66	66.66	0	100									

~~~~~~~~~~ ~~~~~~~~~~ ~~~~~~~~~~ ~~~~~~~~~~ ~~~~~~~~~~ ~~~~~~~~~~

Name: Bernstein 2021 Example 7. Stimulus Number: 1 out of 1 stimuli.
Stimulus: The square peg will settle in the round hole
Response: walk by the peg if it were

DH	AH	S	K	W	EH	R	_	P	EH	G	W	IH
W	AO	_	K	B	AY	DH	AH	P	EH	G	_	IH
L	S	EH	T	AH	L	IH	N	DH	AH	R	AW	N
F	_	_	_	_	_	IH	T	W	ER	_	_	_
D	HH	OW	L									
_	_	_	_									

AH	AW	D	DH	EH	G	HH	IH	K	L	N	OW	P
0	0	0	0	50	100	0	100	100	0	0	0	100
R	S	T	W									
0	0	0	0									

~~~~~~~~~~ ~~~~~~~~~~ ~~~~~~~~~~ ~~~~~~~~~~ ~~~~~~~~~~ ~~~~~~~~~~

Name: Bernstein 2021 Example 8. Stimulus Number: 1 out of 1 stimuli.
Stimulus: The square peg will settle in the round hole
Response: square pig near the round hole

DH	AH	S	K	W	EH	R	P	EH	G	W	IH	L
_	_	S	K	W	EH	R	P	IH	G	N	IH	R
S	EH	T	AH	L	IH	N	DH	AH	R	AW	N	D
_	_	_	_	_	_	_	DH	AH	R	AW	N	D
HH	OW	L										
HH	OW	L										

AH	AW	D	DH	EH	G	HH	IH	K	L	N	OW	P
50	100	100	66.66	50	100	100	50	100	50	50	100	100
R	S	T	W									
80	66.66	0	66.66									

~~~~~~~~~~ ~~~~~~~~~~ ~~~~~~~~~~ ~~~~~~~~~~ ~~~~~~~~~~ ~~~~~~~~~~

Name: Bernstein 2021 Example 9. Stimulus Number: 1 out of 1 stimuli.
Stimulus: The store was jammed before the sale could start
Response: the door will close before the sale is

DH	AH	S	T	AO	R	W	AA	Z	JH	_	AE	M
DH	AH	_	D	AO	R	W	IH	L	K	L	OW	S
D	B	IH	F	AO	R	DH	AH	S	EY	L	K	UH
_	B	IH	F	AO	R	DH	AH	S	EY	L	_	_
D	S	T	AA	R	T							
_	_	_	IH	Z	_							

AA	AE	AH	AO	B	D	DH	EY	F	IH	JH	K	L
0	0	100	100	100	0	100	100	100	50	0	0	50
M	R	S	T	UH	W	Z						
0	80	40	0	0	100	0						

~~~~~~~~~~ ~~~~~~~~~~ ~~~~~~~~~~ ~~~~~~~~~~ ~~~~~~~~~~ ~~~~~~~~~~

Name: Bernstein 2021 Example 10. Stimulus Number: 1 out of 1 stimuli.
Stimulus: The store was jammed before the sale could start
Response: twelve stories can reduce the sale

_	DH	AH	_	_	S	T	AO	R	W	AA	Z	JH
T	W	EH	L	V	S	T	AO	R	_	IY	Z	K
AE	M	D	B	IH	F	AO	R	DH	AH	S	EY	L
AE	N	R	_	IH	D	UW	S	DH	AH	S	EY	L
K	UH	D	S	T	AA	R	T					
_	_	_	_	_	_	_	_					

AA	AE	AH	AO	B	D	DH	EY	F	IH	JH	K	L
0	100	66.66	66.66	0	0	66.66	100	0	100	0	0	66.66
M	R	S	T	UH	W	Z						
0	40	66.66	40	0	0	100						

~~~~~~~~~~ ~~~~~~~~~~ ~~~~~~~~~~ ~~~~~~~~~~ ~~~~~~~~~~ ~~~~~~~~~~

Name: Bernstein 2021 Example 11. Stimulus Number: 1 out of 1 stimuli.
Stimulus: The store was jammed before the sale could start
Response: twelve stories to reduce the pair

_	DH	AH	_	_	S	T	AO	R	W	AA	Z	JH
T	W	EH	L	V	S	T	AO	R	_	IY	Z	T
AE	M	D	B	IH	F	AO	R	DH	AH	S	EY	L
UW	R	_	_	IH	D	UW	S	DH	AH	P	EH	_
K	UH	D	S	T	AA	R	T					
_	_	_	_	_	_	R	_					

AA	AE	AH	AO	B	D	DH	EY	F	IH	JH	K	L
0	0	66.66	66.66	0	0	66.66	0	0	100	0	0	0

M	R	S	T	UH	W	Z
0	66.66	40	33.33	0	0	100

~~~~~~~~~~ ~~~~~~~~~~ ~~~~~~~~~~ ~~~~~~~~~~ ~~~~~~~~~~ ~~~~~~~~~~

Name: Bernstein 2021 Example 12. Stimulus Number: 1 out of 1 stimuli.
Stimulus: The store was jammed before the sale could start
Response: a store induce the sale

DH	AH	S	T	AO	R	W	AA	Z	JH	AE	M	D
_	AH	S	T	AO	R	_	_	_	_	IH	N	D
B	IH	F	AO	R	DH	AH	S	EY	L	K	UH	D
_	_	_	UW	S	DH	AH	S	EY	L	_	_	_
S	T	AA	R	T								
_	_	_	_	_								

AA	AE	AH	AO	B	D	DH	EY	F	IH	JH	K	L
0	0	100	66.66	0	66.66	66.66	100	0	0	0	0	100

M	R	S	T	UH	W	Z
0	50	66.66	50	0	0	0
~~~~~~~~~~
